# Supplementary material for: The maternal genetic make-up of the Iberian Peninsula between the Neolithic and the Early Bronze Age
Source: Sci Rep. 2017 Nov 15;7:15644. doi: 10.1038/s41598-017-15480-9 (PMC5688114; doi:10.1038/s41598-017-15480-9)
Supplement: Supplementary file 1 — Supplementary information and figures [file 41598_2017_15480_MOESM1_ESM.pdf]

The maternal genetic make-up of the Iberian Peninsula between the Neolithic and the Early Bronze Age

Supplementary Figures

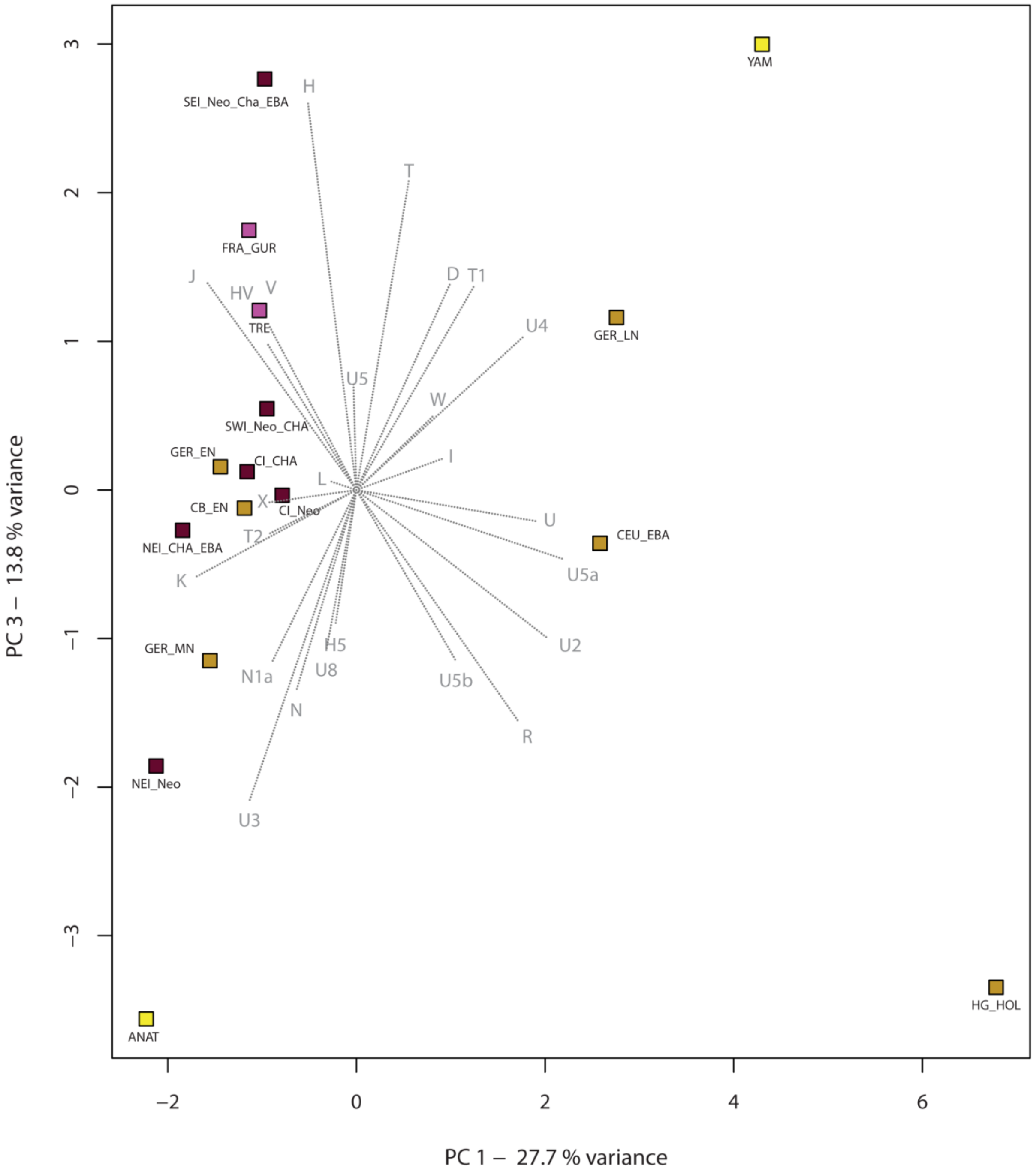

Figure S1. PC1 and PC3 of the PCA of 16 prehistoric groups. The first and third components display 41.5% of the total variance. Altogether haplogroup frequencies of 955 individuals were considered in this analysis. Populations are colored according to their geographical positions: brown: Iberia, purple: France, ochre: Central and East-Central Europe, yellow: Eastern Europe and Near East.

Abbreviations: northeast Iberian Neolithic (NEI_Neo), northeast Iberian Chalcolithic and Early Bronze Age (NEI_CHA_EBA), central Iberian Neolithic (CI_Neo), central Iberian Chalcolithic (CI_CHA), southwest Iberian Neolithic and Chalcolithic (SWI_Neo_CHA), southeast Iberian Neolithic, Chalcolithic and Bronze Age (SEI_Neo_CHA_EBA), Central and North European hunter-gatherers from the Holocene (HG_HOL), Neolithic Anatolia (ANAT) Yamnaya (YAM), Early Neolithic Carpathian Basin (CB_EN), Early Neolithic Germany (GER_EN), Middle Neolithic Germany (GER_MN), Late Neolithic Germany (GER_LN), Early Bronze Age Central Europe (CEU_EBA), Neolithic Gurgy site in France (FRA_GUR), Neolithic Treilles culture in France (TRE). For further information see Supplementary Table S6.

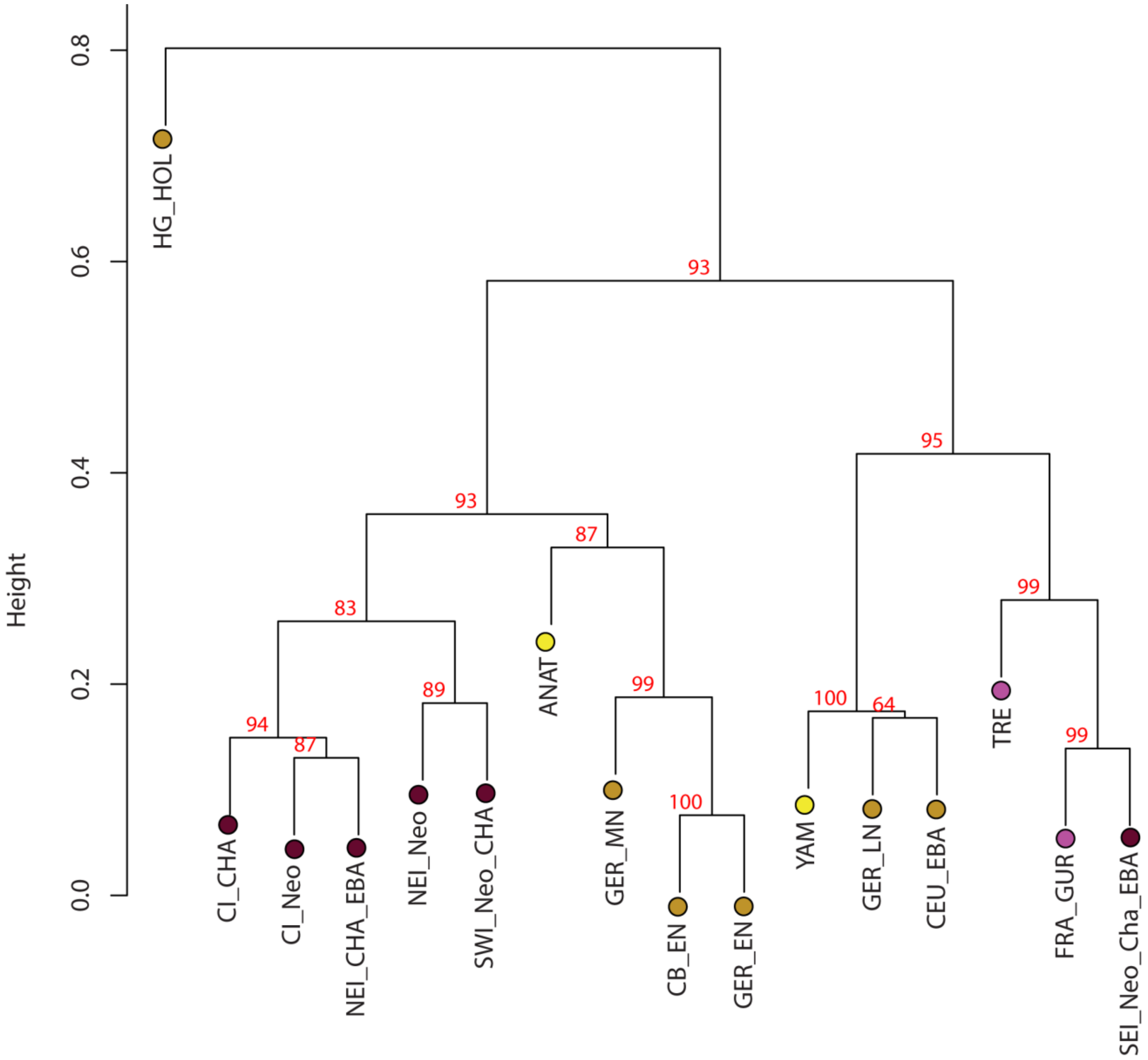

Figure S2. Ward clustering tree of 16 prehistoric groups. The dendogram is based on the frequency of the same 25 haplogroups as used in the PCA. Red numbers indicate p values of the branches in percent. For abbreviations and details on the populations see Figure S2 and Supplementary Table S6.

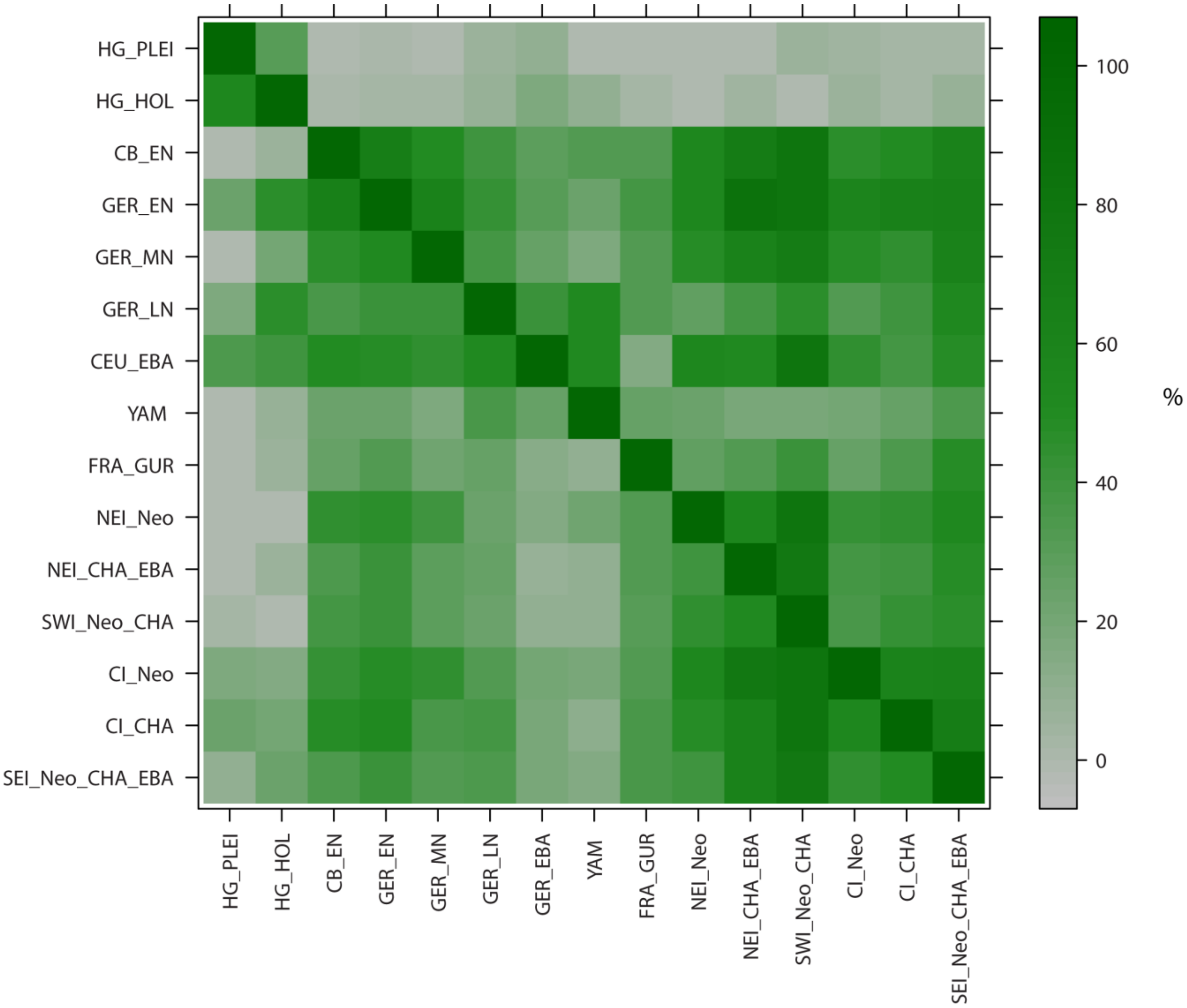

Figure S3. Levelplot of the percentages of shared haplotypes (SHA) between the Iberian and other European prehistoric populations. Principals of the SHA: the HVS-I lineages of the vertically aligned groups were searched in the horizontally listed groups, and the number of total matches per pair of groups were divided by the number of individuals in the horizontally listed groups. Abbreviations: Hunter-gatherers in Central Europe from Pleistocene (HG_PLEI), Hunter-gatherers in Central Europe from the Holocene (HG_HOL), Early Neolithic in the Carpathian Basin (CB_EN), Early Neolithic in Germany (GER_EN), Middle Neolithic in Germany (GER_MN), Early Bronze Age in Central Europe (CEU_EBA), Yamnaya in Eastern Europe (YAM), Gurgy Neolithic site in France (FRA_GUR), northeast Iberian Neolithic (NEI_Neo), northeast Iberian Chalcolithic and Early Bronze Age (NEI_CHA_EBA), southwest Iberian Neolithic and Chalcolithic (SWI_Neo_CHA), central Iberian Neolithic (CI_Neo), central Iberian Chalcolithic (CI_CHA), southeast Iberian Neolithic, Chalcolithic and Early Bronze Age (SEI_Neo_CHA_EBA). For exact values see Supplementary Table S10.

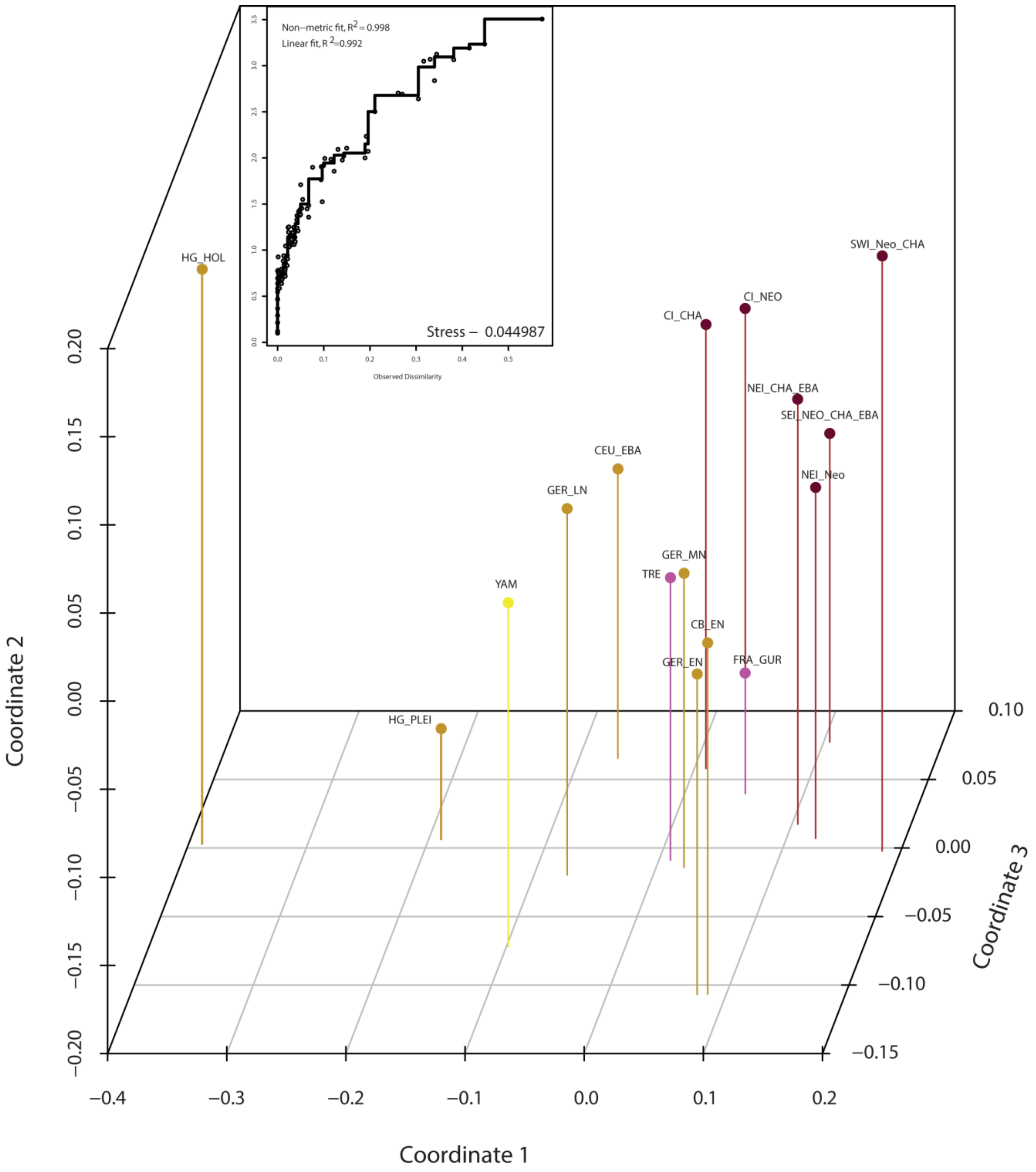

Figure S4. Multidimensional scaling of the Slatkin F_{ST} values, counted between 16 prehistoric groups. The data are presented in 3D. Shepard plot is in the upper left corner, representing the degree of correspondence (stress) and goodness of fit. Populations are colored according to geographical positions: brown: Iberia, purple: France, ochre: Central and East-Central Europe, yellow: Eastern Europe. For abbreviations and population information see Supplementary Table S11.

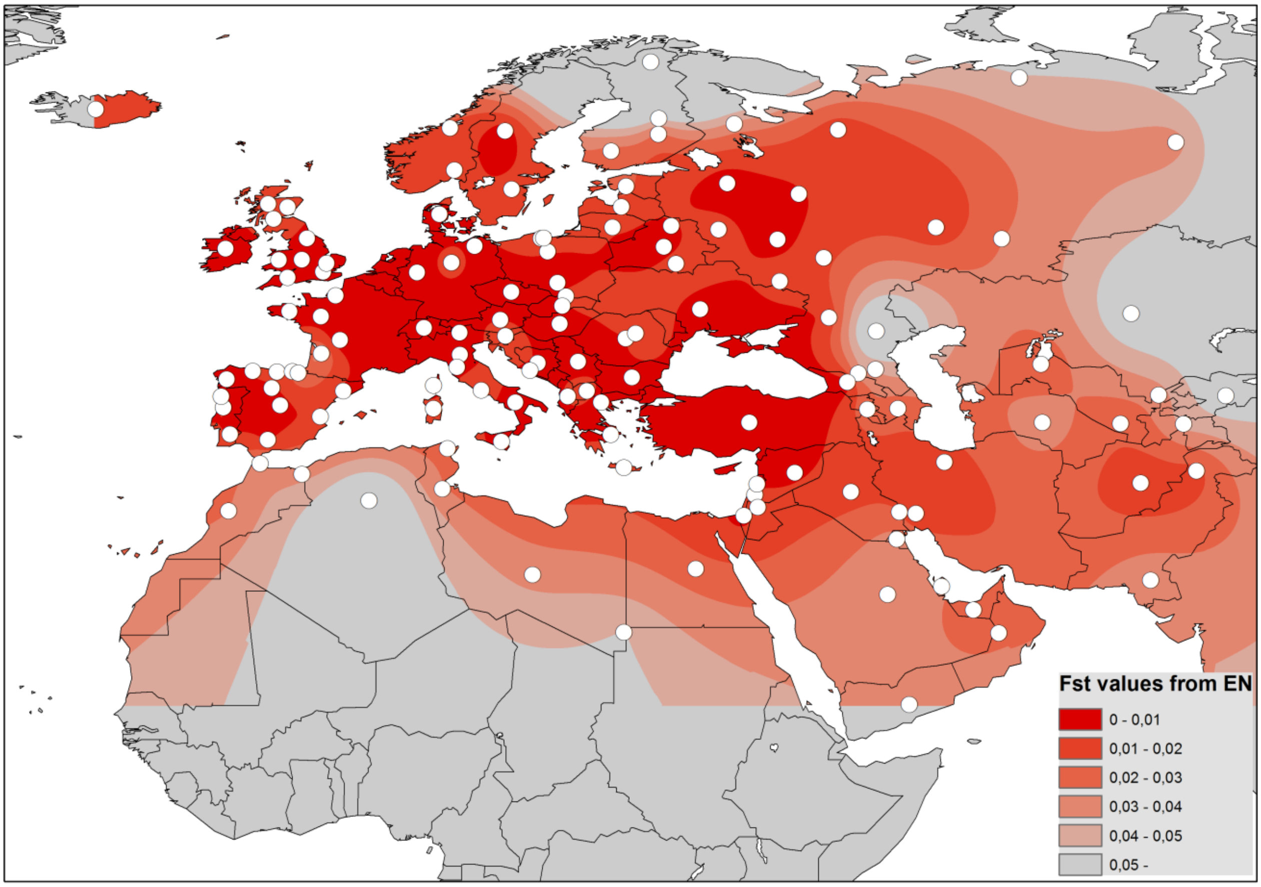
Fst values from EN
0 - 0,01
0,01 - 0,02
0,02 - 0,03
0,03 - 0,04
0,04 - 0,05
0,05 -

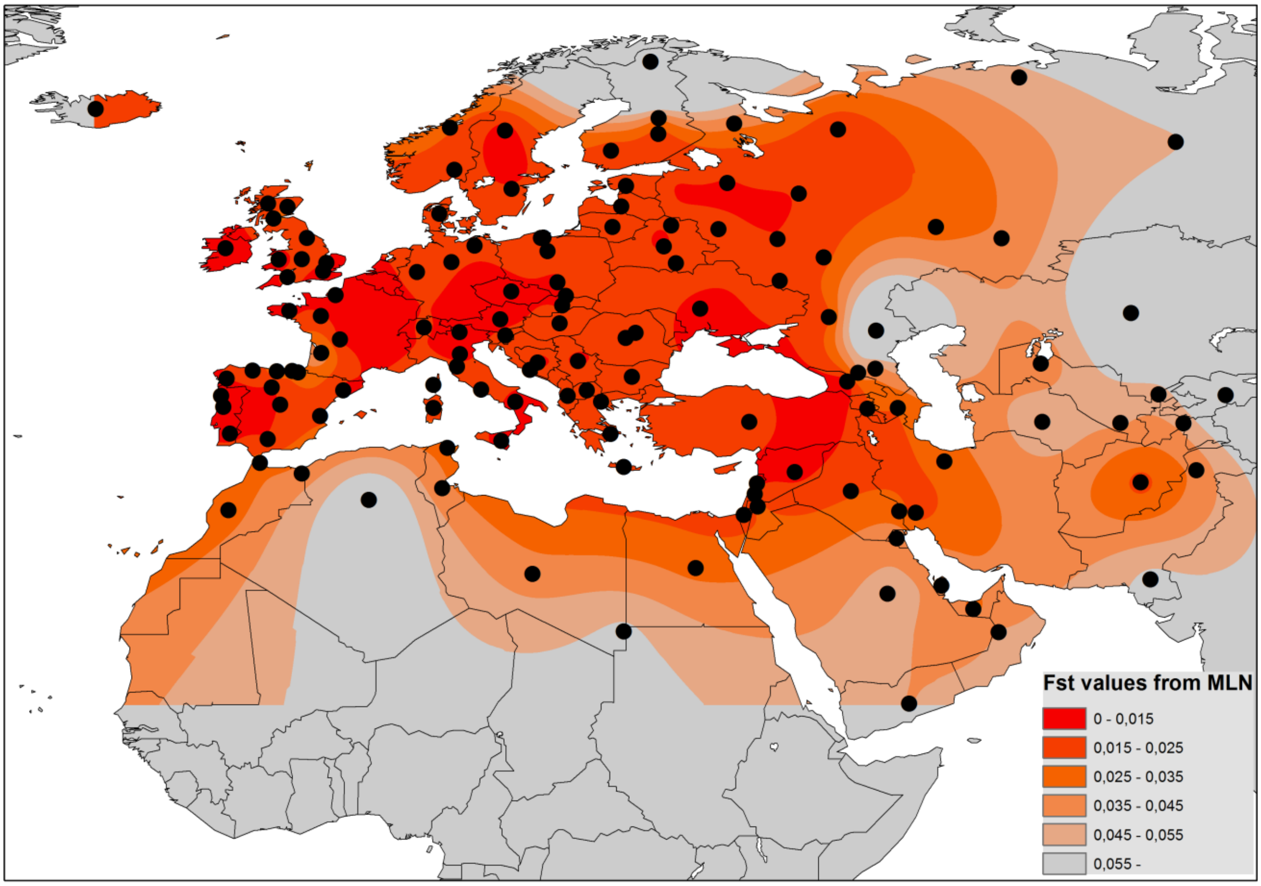
Fst values from MLN
0 - 0,015
0,015 - 0,025
0,025 - 0,035
0,035 - 0,045
0,045 - 0,055
0,055 -

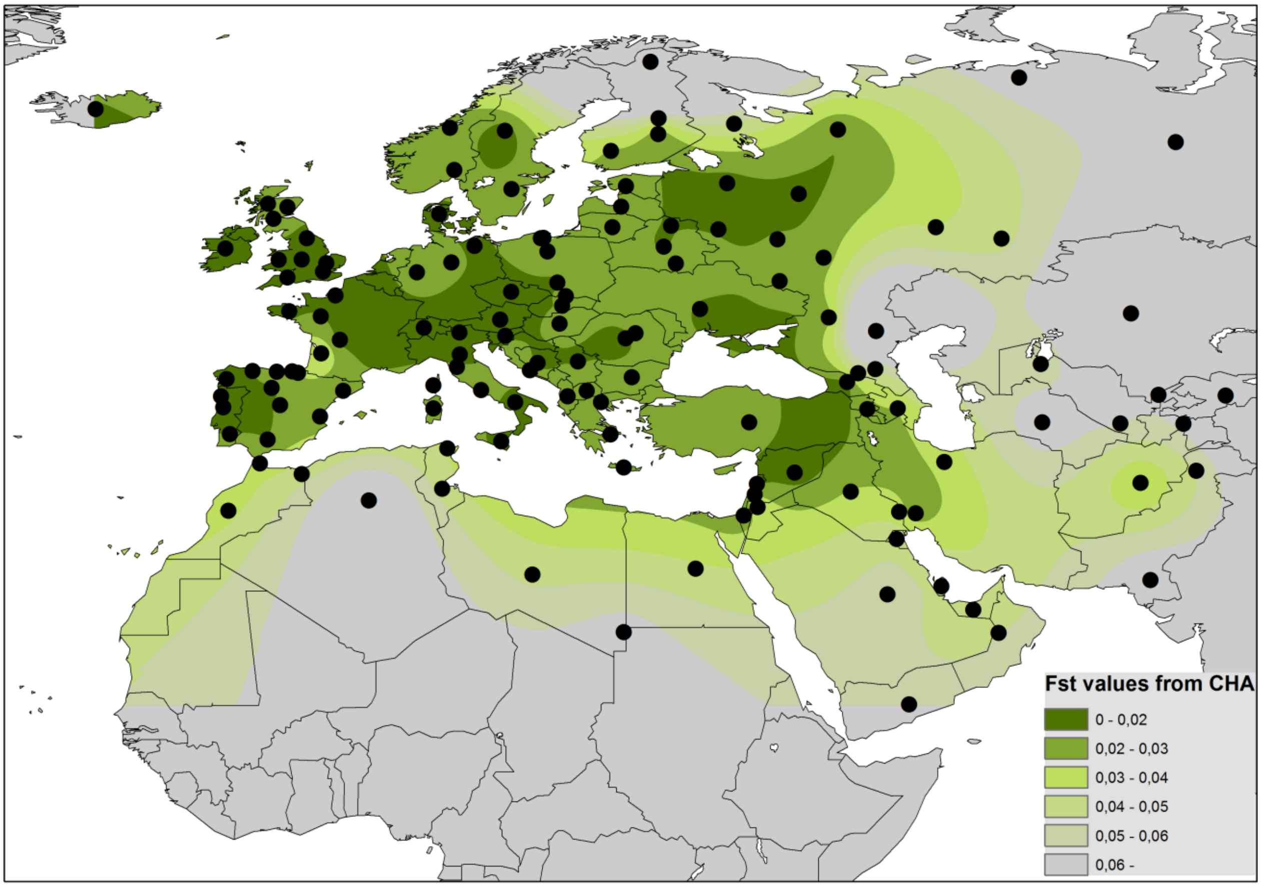

Figure S5. Genetic distance maps. HVS-I sequence based F_{ST} values were counted from Iberian Early Neolithic (A), Middle-Late Neolithic (B), and Chalcolithic (C) populations. Circles signalize the sampled and compared 133 modern populations. Grey regions display F_{ST} values greater than 0.05 (A) or 0.055 (B), 0.06 (C) or unconsidered territories. The values of genetic distances are listed in Supplementary Table S13. The F_{ST} values and coordinates were interpolated with Kriging method implemented in Arcmap ArcGIS version 10.3 (https://www.arcgis.com).

List of Supplementary Tables

Table S1. List of sampled archaeological sites.

Table S2. Sample specific data.

Table S3. MtDNA haplogroup, HVS-I, HVS-II haplotype results.

Table S4. Multiplex SNP typing.

Table S5. Studied sites, grouped by chronological and geographical data.

Table S6. Principal component analyses and Ward type clustering.

Table S7. Fisher's exact test p values.

Table S8. Test of population continuity.

Table S9. Summary statistics.

Table S10. Shared haplotype analyses and ancestral shared haplotypes.

Table S11. F_{ST} values, Slatkin F_{ST} values and p values.

Table S12. Results of AMOVA (analysis of molecular variance) of the Iberian prehistoric groups.

Table S13. F_{ST} values comparing prehistoric Iberia with modern Eurasian populations.

Table S14. HVS-I and HVS-II haplotypes of the contributor researchers.

Table S15. Haplotype and haplogroup information of all published ancient samples used in the study.

Table S16. Comparison of NGS and PCR based haplotype and haplogroup data.

Supplementary Text

Description of the studied archaeological sites

All sites that gave aDNA results are described in alphabetical order. Tables after each site contain those samples that gave reproduced mtDNA data. All dates are calibrated with the Calib Rev. 7.1 program and the IntCal13 calibration curve (Reimer *et al*. 2013).

Reference:
-Reimer, P.J., Bard, E., Bayliss, A., Beck, J.W., Blackwell, P.G., Bronk Ramsey, C., Buck, C.E., Cheng, H., Edwards, R.L., Friedrich, M., Grootes, P.M.: Guilderson, T.P., Haflidason, H., Hajdas, I., Hatté, C., Heaton, T.J., Hogg, A.G., Hughen, K.A., Kaiser, K.F., Kromer, B., Manning, S.W., Niu, M., Reimer, R.W., Richards, D.A., Scott, E.M., Southon, J.R., Turney, C.S. M., Van Der Plicht, J. (2013). IntCal13 and MARINE13 radiocarbon age calibration curves 0-50000 years cal BP. *Radiocarbon*, 55, 4: 1869-1887.

Alto de Rodilla (Monasterio de Rodilla, Burgos, Spain)

Alonso Fernández, C. & Jiménez Echevarría, J.

The archaeological site of Alto de Rodilla was excavated in 2005 by Cronos SC Arqueología y Patrimonio, under the direction of Phd Carmen Alonso. Geographically it is located in the natural corridor that connects the Meseta and the high basin and half of the Valle del Ebro. The excavated evidence (Alonso & Jiménez, 2015) are 20 pits, among them pits-dumps and post holes. Three pits belong to a nearby settlement of the Iron Age and the rest to the Early Neolithic settlement. The pit E-2 contained a child individual of about 10 years old. The pit had an oval form of 85 cm of diameter and cylindrical section. The child was placed in a fetal position facing south-east. Partial dislocation of the remains appears to indicate empty decomposition. The body could be placed sitting and slightly lying on the left side. The pit was first sealed with stones. On them were small fragments of pottery (one decorated with boquique technique), a quartz hexagonal prism and a quartzite polisher. The pit was finally filled with ash and burned stones that had some ceramic fragments and sheep-goat bones. A radiocarbon date from a human bone places the burial at the last quarter of the 6th millennium cal BCE, culturally in the Early Neolithic.

SITE	SAMPLE NUMBER	ARCHAEOLOGICAL CONTEXT	LAB ID	DATE BP	CALIBRATED DATA 2σ	AGE/SEX	PERIOD
Alto de Rodilla	Rodi1	E2	CSIC-1967	6170±55	5295-4986 BCE	Child	Early Neolithic

References:
- Alonso Fernández, C. & Jiménez Echevarría, J., 2015. El Neolítico en el corredor Alto Ebro-Alto Duero: dos hallazgos funerarios del Neolítico Antiguo y Reciente en Monasterio de Rodilla (Burgos), *5.º Congresso do Neolítico Peninsular*, UNIARQ, Lisboa, pp. 540-546.

Arroyal I (Alfoz de Quintanadueñas, Spain)

Carmona, E. & Arnaiz, M.A.

The site of Arroyal I was excavated by a research team from the University of Burgos in 2011-2012. The site is a megalithic grave with well-preserved structural elements: a rectangular chamber (3 x 3.5 m), a long corridor (6 m) and a stone mound. The grave was used as a collective burial during 400 years in the Late Neolithic (3300-2900 cal BCE) (Carmona Ballestero & Arnaiz Alonso, 2015). Then the grave was abandoned, until the Chalcolithic when it was extensively remodeled: Neolithic layers were almost eliminated, the corridor was filled with rocks and sediment (a clear closure); the useful area inside the chamber was reduced when a stone wall was built; and a floor of limestone blocks was built inside the chamber. Then, several consecutive and isolated burials (9-10) were introduced. The last one was a young individual (UGA-15903) buried with a set of 4 vessels (2 Bell Beakers and 2 carinated bowls), and surrounded by the long bones and skulls from previous burials. Then the dolmen was closed using materials from the site (in secondary position) and, at the same time, the mound height was increased. Finally, an isolated pit grave was made inside the mound (MAMS-14857).

SITE	SAMPLE NUMBER	ARCHAEOLOGICAL CONTEXT	LAB ID	DATE BP	CALIBRATED DATA 2σ	PERIOD
Arroyal I	Roy4	UE 19 inhumation 1	MAMS-14857	3837±25	2455-2201 BCE	Late Copper Age
Arroyal I	Roy5	UE 25 inhumation 2	UGA-15903	3870±30	2464-2212 BCE	Late Copper Age
Arroyal I	Roy1	UE25 skull1	UGA-15904	3850±30	2458-2206 BCE	Late Copper Age
Arroyal I	Roy3	UE 25 skull 2	UGA-15905	3860±30	2461-2209 BCE	Late Copper Age
Arroyal I	Roy2	UE 25 Mand 1	-	-	-	Late Copper Age?

References:
- Carmona Ballestero, E. & Arnaiz Alonso, M.A., i.p. Stratigraphic events and 'Cycles of use' in the dolmen of Arroyal I. In M.A. Rojo Guerra & C. Tejedor-Rodríguez, eds. *Biografías Megalíticas. Proceeddings of XVII World UISPP Congress*, In press.

Balma Sargantana (Oliola, Lleida, Spain)

Soriano, I. & Oliart, C.

This funerary site was excavated 1979, 1983 and 1984 and has been defined as a para-dolmenic structure or a cave-dolmen. These caves or rock shelters with modified pavements, closing walls and large stone slabs, which divide the inner space, imitate megalithic monuments, which are contemporary. In this case, a natural rock shelter dug into the clay was excavated in order to enlarge the inner space (some 3 m in length), which had been paved and sub-divided by three perpendicularly placed slabs. Slabs must have blocked the structure, as marks in the floor suggest. The interments (MNI=52) form a unique stratum of successive, primary burials. Bodies were displaced and bones (skulls, long bones) were grouped together in heaps. The anthropological study has identified two trepanation and possible evidences of violence. Among the recovered materials are a copper awl, 7 flint blades, 7 flint arrowheads, two plain hemispherical bowls and abundant ornamental beads made of bone and stone (Petit, 2001). Although we do not dispose of ^{14}C dates, this type of burials can be documented during Early and Late Copper Age of northeast Iberia (c. 3400 – 2300 cal BCE) (Soriano, 2013; 2016).

SITE	SAMPLE NUMBER	ARCHAEOLOGICAL CONTEXT	PERIOD
Balma de Sarganta	Bas3	BS-C3-1154	Copper Age
Balma de Sarganta	Bas5	BS-D3,2e 159	Copper Age

References:
- Petit, Mª A., 2001. Els primers pagesos i ramaders. De la 2a meitat del VI mil·lenni a mitjan del III mil·lenni cal BCE. *La Noguera antiga. Des dels primers pobladors fins als visigots*: 46-61. Museu d'Arqueologia de Catalunya, Museu de la Noguera, Ajuntament de Balaguer, Girona.
- Soriano, I. 2013. Metalurgia y sociedad en el nordeste de la Península Ibérica (finales del IV-II milenio cal ANE). *Oxford, Archaeopress, British Archaeological Reports International Series, 2502.*
- Soriano, I. 2016. Les pràctiques funeràries durant el Calcolític i Bronze Antic i Mig. In J. Bosch, M. Borrell, ed. *La fi és el Principi. Pràctiques funeràries a la Catalunya prehistòria. Catàleg de l'exposició.* Museu d'Arqueologia de Catalunya, pp. 77-97.

Barranc d'en Rifà (Mont-roig del Camp, Tarragona, Spain)

Soriano, I. & Oliart, C.

This funerary site was discovered in 1971 during the construction of a water channel for the nuclear power station of Vandellós. The news spread and lead to illegal excavations before the systematic exploration started. The burial, probably a hypogeum, opened on the left margin of the d'en Rifà ravine. Among the recovered materials are a copper awl, 11 v-perforated buttons, pyramidal bone buttons, two plain hemispherical bowls and a series of human bones, which have not yet been analysed systematically (Vilaseca 1973, 190-192). Two radiocarbon dates analysed from two different individuals in the frame of this project place the burial to the Early Coper Age of northeast Iberia (4138±26 BP, 4217±25 BP).

SITE	SAMPLE NUMBER	ARCHAEOLOGICAL CONTEXT	LAB ID	DATE BP	CALIBRATED DATA 2σ	PERIOD
Barranc d'en Rifà	BAR1	BR14	MAMS-14854	4138±26	2872-2622 BCE	Early Copper Age
Barranc d'en Rifà	BAR2	BR17	MAMS-14855	4217±25	2899-2700 BCE	Early Copper Age
Barranc d'en Rifà	BAR3	BRG	-	-	-	Early Copper Age
Barranc d'en Rifà	BAR4	BRH	-	-	-	Early Copper Age
Barranc d'en Rifà	BAR5	BRI/L	-	-	-	Early Copper Age
Barranc d'en Rifà	BAR6	BRG	-	-	-	Early Copper Age
Barranc d'en Rifà	BAR7	BRM	-	-	-	Early Copper Age
Barranc d'en Rifà	BAR8	BR10	-	-	-	Early Copper Age
Barranc d'en Rifà	BAR9	BR19	-	-	-	Early Copper Age
Barranc d'en Rifà	BAR10	BR-CRO1	-	-	-	Early Copper Age
Barranc d'en Rifà	BAR11	BR-CRO2	-	-	-	Early Copper Age
Barranc d'en Rifà	BAR12	BR4	-	-	-	Early Copper Age

Barranc d'en Rifà	BAR13	BR20	-	-	-	Early Copper Age
Barranc d'en Rifà	BAR14	BRC	-	-	-	Early Copper Age
Barranc d'en Rifà	BAR15	BRB	-	-	-	Early Copper Age

References:
- Vilaseca, S., 1973. *Reus y su entorno en la Prehistoria* (2 vol.). Asociación de Estudios Reusenses: 48-49. Ediciones Rosa de Reus, Reus.

Bolores (Torres Vedras, Portugal)

Soriano, I. and Lillios K.

Bolores is an arched-roof rock-cut tomb carved into an outcrop of sandstone, with a shale floor and low walls of interbedded sandstone and shale. It measures 5.6 m north-south by 1.7 m east-west, with a maximum height of 1.5 m. The tomb is located at an elevation of 37-38.5 m above sea level. Following a test season in 1986, an interdisciplinary team of the University of Iowa conducted four campaigns between 2007 and 2012. Eleven AMS radiocarbon dates were obtained from different individuals, recovered at different depths in the mortuary deposit. The majority of them cluster between 2800-2600 cal BCE (Early Copper Age). The architectural, material and bioarchaeological evidence suggest that Bolores housed the remains of a distinctive group of local individuals who marked their difference from other burials populations in the Sizandro and Estremadura region. Social differences were denoted spatially and through offerings (Lillios et al. 2015). Skeletal remains of at least 36 individuals were found, both males and females: 19 adults, 4 adolescents and 13 children (Mack et al. 2016). Analyses of the strontium isotopes ratios (87Sr and 86Sr) of 19 individuals revealed that they all appear to have spent their lives in the local geological region. Some bones of the individuals buried were stained with red ochre. Funerary offerings included ceramic bowls, flint blades, a bone point, a bone handle stone and shell beads and stone idols of different morphologies.

SITE	SAMPLE NUMBER	ARCHAEOLOGICAL CONTEXT	LAB ID	DATE BP	CALIBRATED DATA 2σ	AGE/SEX	PERIOD
Bolores	BOL.11.N4	Individual 11N.1; Adult 2	Beta-249032	4150±40	2879-2589 BCE	Adult	Early Copper Age

References:
- Lillios, K.T., Mack, J., Waterman, A.J., Artz, J.A, and Nilsson-Stutz, L., 2015. *In Praise of Small Things: Excavations at the Late Neolithic-Early Bronze Age Burial of Bolores (Torres Vedras), Portugal*. British Archaeological Reports, International Series. Oxford.
-Mack, J., Waterman, A.J., Racila, A.-M., Artz, J.A., and Lillios, K.T. 2016. Applying zooarchaeological methods to interpret mortuary behavior and taphonomy in commingled burials: The case study of the Late Neolithic site of Bolores, Portugal. *International Journal of Osteoarchaeology* 26(3), pp. 524-536. 10.1002/oa.2443.

Cabezo Pardo (San Isidro-Granja de Rocamora, Alicante, Spain)

López Padilla, J.A. & Jover Maestre, F. J.

The site of Cabezo Pardo is an Early Bronze Age settlement of the Argar Culture. It is located on a hilltop in the lower Valley of Segura River. Its discovery dates back to the early twentieth century, although it was not systematically excavated until 2006. It is of small size (ca. 0.25 ha) and mainly oriented to the farm, hunting and fishing in the gaps in their environment. After an initial phase of occupation dated around 1950 cal BCE, with more or less isolated houses on the hilltop, the urban plan of the settlement changed completely around 1800 cal BCE. In this second phase a central building with thick walls was built on top of the hilltop, surrounded by a narrow street that connected with a series of arranged radially houses. The only two graves found at the site were constructed at this phase. Around 1500 cal BCE there appears to be the definitive abandonment of the settlement.

SITE	SAMPLE NUMBER	ARCHAEOLOGICAL CONTEXT	LAB ID	DATE BP	CALIBRATED DATA 2σ	AGE/SEX	PERIOD
Cabezo Pardo	Cab1	Grave 1 (bone 10)	Beta-237765	3460±40	1889-1668 BCE	Adult male	Early Bronze Age
Cabezo Pardo	Cab2	Grave 1 (bone 54)	Beta-237766	3390±40	1870-1560 BCE	Adult female	Early Bronze Age

References:
- López Padilla, J.A. et al., 2015. El registro funerario de Cabezo Pardo (San Isidro/ Granja de Rocamora, Alicante) MARQ. Arqueología y Museo 06, pp. 123-145.

Camino de las Yeseras (San Fernando de Henares, Madrid, Spain)

Liesau, C., Rios, P. & Blasco, C., Vega, J.& Menduiña, R.

Camino de Las Yeseras in the Northeast of Madrid is a c. 22 ha large Chalcolithic ditched enclosure site starting at the beginning of the 3rd millennium BCE. It has a long time occupation with Bell Beaker and non-Bell Beaker structures as also a reduced Bronze Age settlement. The site probably was a central place, as can be deduced from its size, its strategic location and the good natural communication routes in a place in which most basic resources were available such as pasture land, flint, clay, salt. The architecture is complex with almost five concentric ditched enclosures, several huts with sunken floors and large numbers of pits and burials (Blasco et al., 2009; 2011, Liesau et al., 2008; Ríos, 2011). The site reveals also interesting faunal deposits in pits, graves and ditches that suggest a complex symbolic relationship of the inhabitants with animals since the middle of the 3rd millennium cal BCE. Camino de las Yeseras is one of the central Iberian chalcolithic sites, in which different burial traditions could be studied and more than 60 individuals have been retrieved (Blasco et al., 2011; Gómez et al., 2011). From the first half of the 3rd millennium BCE, several collective inhumations in pits are the most common burial features. For the second half of the 3rd millennium BCE, other contemporaneous non-Bell Beaker burials are still present when a Bell Beaker occupation (2450-1740 cal BCE), has been documented. The individualization tendency in the funerary practices might reveal social changes where single or double inhumations delimited in special funerary areas (small artificial caves and deep hypogea) reveal new burial customs. The exclusiveness of these burials is not only characterized by a complex burial architecture but by several other items as Bell Beaker ware with symbolic decoration, copper artifacts, gold and ivory ornaments, some of them testifying long distance exchanges (Blasco et al., 2011; Liesau y Moreno, 2012).

SITE	SAMPLE NUMBER	ARCHAEOLOGICAL CONTEXT	LAB ID	DATE BP	CALIBRATED DATA 2σ	PERIOD
Camino de las Yeseras	Yese1	Area 35 El03	-	-	-	Late Copper Age
Camino de las Yeseras	Yese2	Area 10 UE 03 E01 Ind 2			2031-1948 BCE	Late Copper Age
Camino de las Yeseras	Yese3	Area 10 UE 05 E07 Ind 1			2467-2346 BCE	Late Copper Age
Camino de las Yeseras	Yese4	Area 10 E01 Ind 1	-	-	-	Late Copper Age
Camino de las Yeseras	Yese5	Area 36 E02 Ind 2			1955-1781	Late Copper Age
Camino de las Yeseras	Yese6	Area 85 E02 Ind 2			2299-2202 BCE (ind 4)	Late Copper Age
Camino de las Yeseras	Yese7	Area 10 UE 05 E07 Ind 8			1921-1776 BCE	Late Copper Age
Camino de las Yeseras	Yese8	Area 10 E07 Ind 7	-	-	-	Copper Age
Camino de las Yeseras	Yese9	Area 85 E02 Ind 5			2299-2202 BCE (ind 4)	Late Copper Age
Camino de las Yeseras	Yese10	Area 10 UE 05 E07 Ind 3	-	-	-	Copper Age
Camino de las Yeseras	Yese12	Area 31 UE 09 Ind 1-I	-	-	-	Late Copper Age
Camino de las Yeseras	Yese13	Area 10 UE 05 E07 Ind 4	-	-	-	Copper Age

References:
- Blasco, C. et Al., 2009. Kupferzeitliche Siedlungsbestattungen mit Glockenbecher- und Prestigebeigaben aus dem Grabenwerk von El Camino de las Yeseras (San Fernando de Henares, prov. Madrid). Untersuchungen zur Typologie des Grabritus und zu dessen sozialer Symbolik. *Madrider Mitteilungen,* 50, 2011, pp. 40-70.
- Blasco, C., Liesau, C. & Ríos, P., 2011. *Yacimientos calcolíticos con campaniforme de la región de Madrid: Nuevos estudios. Patrimonio arqueológico de Madrid*, 6. Universidad Autónoma de Madrid.
-Gómez Pérez, J.L. et al., 2011. Los protagonistas. In C. Blasco, C. Liesau & P. Ríos, eds. Patrimonio Arqueológico de Madrid, 6. UAM, pp. 100-132.
- Liesau, C. et al., 2008. Un espacio compartido por vivos y muertos: el poblado calcolítico de fosos de Camino de las Yeseras. *Complutum*, 19 (1), pp. 97-120.
- Liesau, C. & Moreno, E., 2012. Marfiles campaniformes de El Camino de las Yeseras (San Fernando de Henares, Madrid). In A. Banerjee, J. A. López Padilla,Th. X. Schuhmacher, eds. *Marfil y Elefantes en la Península Ibérica y el Mediterráneo,* Internationale Tagung. Iberia Archeologica, 16,1, pp. 83-94.
- Ríos, P., 2011. *Territorio y Sociedad en la Región de Madrid durante el III milenio a. C. El referente del yacimiento de Camino de las Yeseras*. Col. Patrimonio Arqueológico de Madrid, 7. Madrid: Ed. Dpto. Prehistoria y Arqueología - UAM (digital).

Camino del Molino (Murcia, Spain)

Lomba, J. & Avilés Fernández, A.

The site Camino del Molino was excavated by the University of Murcia in 2008. It is a multiple burial with remains of 1363 individuals, deposited in a circular pit of 7 m of diameter and 1.6 m of depth, excavated in the rock. It’s located to 500 m of the habitat with which a settlement seems to be related, Molinos de Papel, with huts and silos also excavated in the soil. The majority of the human remains appear in chaotic position, fruit of continuous postdepositional movements during the use of the grave, replacing many of them in the center of the pit, with accumulations of skulls across the walls. 182 cases remain in anatomical complete or partial position, normally in foetal position, one

individual was placed mouth below with the hands tied to the back. The study of skulls and these 182 skeletons indicates equal proportion of men and female (49.5 and 44.8 %), of which 47.5 % are young, 33.6 % mature and 4.5 % senile; in 20 skulls interpersonal violence was detected (57% males). Accompanying the human remains there were 20 copper elements (19 punches, a fragment of a Palmela point and a dagger), 40 arrowheads and 60 flint knifes (fragmented or not), 4 polished axes, retouched tabular flint (among them several daggers) and some punches and rods made of bone, as well as partial remains of approximately 400 pottery vessels, 30 of which were complete. Of special interest it is the presence of 50 skeletons of canis (6 lupus and the rest familaris). The whole material is assigned to a Middle and Final Copper Age, coinciding with the funeral modality. The 20 radiocarbon dates show a use sequence of the tomb between 2920-2870/2800-2780 and 2460-2190/2180-2140 cal BCE (2 sigma).

SITE	SAMPLE NUMBER	ARCHAEOLOGICAL CONTEXT	LAB ID	DATE BP	CALIBRATED DATA 2σ	PERIOD
Camino del Molino	CMOL 6	CMOL6	Beta- 261517	3910±40	2550-2230 BCE	Late Copper Age
Camino del Molino	CMOL 12	CMOL12	-	-	-	Late Copper Age
Camino del Molino	CMOL 16	CMOL16	Beta- 261519	3970±40	2580-2345 BCE	Late Copper Age
Camino del Molino	CMOL 44	CMOL44	-	-	-	Copper Age
Camino del Molino	CMOL 71	CMOL71	-	-	-	Copper Age
Camino del Molino	CMOL 79	CMOL79	Beta- 261524	3830±40	2460-2145 BCE	Late Copper Age
Camino del Molino	CMOL 104	CMOL104	-	-	-	Copper Age
Camino del Molino	CMOL 105	CMOL105	Beta- 261527	4130±40	2875-2580 BCE	Early Copper Age
Camino del Molino	CMOL 116	CMOL119	-	-	-	Copper Age
Camino del Molino	CMOL 122	CMOL122	-	-	-	Copper Age
Camino del Molino	CMOL 123	CMOL123	Beta- 261529	4210±40	2905-2665 BCE	Early Copper Age
Camino del Molino	CMOL 138	CMOL138	-	-	-	Copper Age
Camino del Molino	CMOL 139	CMOL139	-	-	-	Copper Age
Camino del Molino	CMOL 140	CMOL140	-	-	-	Copper Age
Camino del Molino	CMOL 151	CMOL151	-	-	-	Copper Age
Camino del Molino	CMOl 154	CMOL154	-	-	-	Copper Age
Camino del Molino	CMOl 164	CMOL164	-	-	-	Copper Age
Camino del Molino	CMOl 165	CMOL165	-	-	-	Copper Age

References:
- Lomba, J., et al., 2009. El enterramiento múltiple, calcolítico, de Camino del Molino (Caravaca, Murcia). Metodología y primeros resultados de un yacimiento excepcional. *Trabajos de Prehistoria,* 66(2), pp. 143-159.

Can Gambús-1 (Sabadell, Barcelona, Spain)

Roig Buxó, J.

The archaeological site of Can Gambús is one of the most important prehistoric landscapes in the Iberian Peninsula, with more than a thousand archaeological structures dating between the middle Neolithic and the Iron Age. The site was excavated between 2003-2006 by Arrago SL, under scientific direction of Jordi Roig and Joan Manel Coll (Roig & Coll, 2007, 2010; Roig et al., 2010). About fifty archaeological structures belong to the Middle Bronze Age (1850-1200 cal BCE), three of them being complex funerary structures. These chambers with an entrance shaft with multiple and successive inhumation are scattered over the hill of Can Gambús and they are associated to storage structures and large half-sunken-huts. Most of the structures are, however, storage pits, followed by large cuts and underground basins that are likely to be attributable to activity and/or domestic areas. Abundant hand-made pottery with characteristic plastic decoration allows to place these structures within the Middle Bronze Age. Radiocarbon dates are being measured at the moment. The human samples analysed in this study come, in the first place, from structure E172, consisting of a reused storage pit, transformed into an access well with two lateral sepulchral cavities. Only one individual was buried inside cave 1, whilst six of them were found in cave 2. The second sampled complex funerary structure is E238, which is located on the east side of the ridge, opposed to the structure E172. In this case, it is also associated with a small group of storage pits. Chamber with an entrance shaft E238, unlike the previous one, consists of a cylindrical access pit with a single lateral cavity where four individuals were buried (three females and one indeterminate). At the bottom of the access pit a bundle of bones corresponding to a male individual. Finally, structure E349 is located at the top of the Can Gambús hill. It consists of a rectangular, nearly one meter deep access pit with two lateral burial caves. In the southern cavity there was an individual in anatomical connection and a bone package corresponding to other individuals, which were scattered along the funerary chamber. On the other hand, the northern cavity presented a large bone package corresponding to several disarticulated skeletons.

SITE	SAMPLE NUMBER	ARCHAEOLOGICAL CONTEXT	PERIOD
Can Gambús	GAM1	1074	Bronze Age
Can Gambús	GAM2	1075	Bronze Age
Can Gambús	GAM3	1076	Bronze Age
Can Gambús	GAM4	E-172 UE-650 Ind 1	Bronze Age
Can Gambús	GAM5	E-172 Ue-448	Bronze Age
Can Gambús	GAM6	E-172 UE-655 Ind 6	Bronze Age
Can Gambús	GAM7	E-172 UE-650	Bronze Age
Can Gambús	GAM8	E-349 UE- 1056 Coord. 76	Bronze Age

References:
- Roig, J. & Coll, J.M., 2007. El paratge arqueològic de Can Gambús-1 (Sabadell, Vallès Occidental), *Tribuna d´Arqueologia 2006,* Generalitat de Catalunya, pp. 85-109.
- Roig, J. et al., 2010. La necrópolis de Can Gambús-1 (Sabadell, Barcelona). Nuevos conocimientos sobre las prácticas funerarias durante el Neolítico medio en el Noreste de la Península Ibérica, *Trabajos de Prehistoria*, 67(1), pp. 59-84.
- Roig, J & Coll, J.M., 2010. La necròpolis del Neolític Mitjà de Can Gambús-1 (Sabadell, Vallès Occidental): nova tipologia dels sepulcres de fossa i pràctiques funeràries durant el IV mil.leni Cal BC a Catalunya, *Cypsela* 18, pp. 93-122.

Cobre las Cruces-SE-K (Salteras, Sevilla, Spain) (Fig.1 nº17)

Hunt Ortiz, M.A., Vázquez Paz, J. & Pecero Espín, J.C.

The archaeological site of SE-K was investigated in 2006 as one of the rescue excavations carried out within the planned Las Cruces open pit copper mine, affecting almost 1000 ha (Hunt Ortiz, 2012). The site is located in the fertile agricultural plains of the Guadalquivir river valley, about 20 km to the NW of the city of Seville. Site SE-K is a Bronze Age cemetery formed mainly by cists and circular pits (18 in number). A total of 27 graves were excavated, containing 39 individuals. Human remains in primary position show a crouched position (females on the right side, and males on the left side). Pit grave T-23 was exceptional and contained three simultaneous female inhumations (Hunt Ortiz, 2012). Grave goods, consisting mainly of pottery vessels, copper (or arsenical copper) awls and *Pecten maximus* shells also showed differences related to sex. Radiocarbon dates obtained from teth from inhumations of graves T-23b (pit), T-7 (cist), and T-25 (cist), gave the following results, respectively: 3570±50 BP, 3500±50 BP and 3460±40 BP (Hunt Ortiz *et al.*, 2008).

SITE	SAMPLE NUMBER	ARCHAEOLOGICAL CONTEXT	LAB ID	DATE BP	CALIBRATED DATA 2σ	AGE/SEX	PERIOD
SE-K	Cru2	T-12 pit burial	--	--	--	Adult (18-25 y.)	Bronze Age
SE-K	Cru1	T-12 pit burial	--	--	--	Female (17-21 y.)	Bronze Age
SE-K	Cru4	T-23 pit burial	--	--	--	Female (18-21 y.)	Bronze Age
SE-K	Cru3	T-23 pit burial	BETA-225416	3490±50	1938-1689 BCE	Female (33-45 y.)	Bronze Age

References:
- Hunt Ortiz, M.A. (2012) *Intervenciones Arqueológicas en el Área del Proyecto Minero Cobre Las Cruces (1996-2011): De la Prehistoria a la Época Contemporánea*. Fundación Cobre Las Cruces. Sevilla.
- Hunt Ortiz, M.A., Vázquez Paz, J., García Rivero, D. & Pecero Espín, J.C. (2008) Dataciones radiocarbónicas de las necrópolis de la Edad del Bronce SE-K, SE-B y Jardín de Alá (Términos Municipales de Salteras y Gerena, Sevilla). En: S. Rovira Llorens, M. García-Heras, M. Gener Moret & I. Montero Ruiz (Eds.) *Actas del VII Congreso Internacional de Arqueometría*: 226-234. CSIC. Madrid.

Cova de la Ventosa (Piera, Barcelona, Spain)

Soriano, I. & Oliart, C.

This funerary site was excavated during the 1970's by a hiking group, while the information and materials from the site were deposited at the Museu d'Arqueologia de Catalunya. This para-dolmenic cavity is formed by a rock shelter, which had been closed with a stone wall. Inside, two individuals had been placed lateral position (left and right), opposite of each other, aligned following a north-south axis, with their feet nearly in contact. A Pyrenees-type bell beaker was found close to the skull of one of the individuals, while the other was accompanied only by a fragment of a plain vessel. Both were 18 years old and have been determined as masculine, although only few bone remains are available (Llongueras et al., 1981). This identification is surprising, as similar positions are typical of the central European bell beaker burials, which usually combine a male and a female individual. No

^{14}C dates are available for this site, but Bell Beaker burials can be dated in northeast Iberia between 2750 – 2300 cal BCE (Soriano, 2016).

SITE	SAMPLE NUMBER	AGE/SEX	PERIOD
Cova de la Ventosa	Vent1	Adult male	Late Copper Age

References:

- Llongueras, M. et al., 1981. Enterrament campaniforme a la Cova de la Ventosa (Pierra, Anoia), *Ampurias* 43, pp. 97-111.
- Soriano, I. 2016. Les pràctiques funeràries durant el Calcolític i Bronze Antic i Mig. In J. Bosch, M. Borrell, ed. *La fi és el Principi. Pràctiques funeràries a la Catalunya prehistòria. Catàleg de l'exposició.* Museu d'Arqueologia de Catalunya, pp. 77-97.

Cova del Barranc del Migdia (Xàbia, Alicante, Spain)

Soler Díaz, J.A., Roca de Togores Muñoz, C. & Esquembre Beviá, M.A.

This cave opens on the southern slope of the Serra of Montgó, about 375 m a.s.l. The more accessible of the two entrances has still difficult access as it requires to climb 12 meters height. A narrow corridor of 12 meters length and no more than 1.2 m width gives access to a central hall, or polygonal area of about 14 square meters, where the sunlight only reaches occasionally. After a step fall of about 4 meters, the room leads to an exterior court that ends with a cliff of about 40 meters height and has various rock paintings resembling Schematic Rock Art. In the central hall interdisciplinary archaeological explorations have been carried out between 2009 and 2014 with the sponsorship of the CIRNE Foundation of Xabia and through the collaboration of the Archaeological Museum "Soler Blasco" of Xabia, the MARQ of Alicante and the Arpa Patrimonio Company. Regarding the funerary use of the cave, human remains without any anatomical order were found forming packages. So far we have identified a minimum of 10 individuals. Taking into account the access conditions and the lack of space, they should have reached the cavity in a disarticulated state. The ^{14}C dates obtained from the human remains establishes the funerary use of the cavity around 2650-2250 cal BCE. Among the funerary offerings, flint arrowheads, polished stone tools, plain pottery and a copper awl stands out. No Bell Beaker pottery was observed.

SITE	SAMPLE NUMBER	ARCHAEOLOGICAL CONTEXT	LAB ID	DATE BP	CALIBRATED DATA 2σ	AGE/SEX	PERIOD
Cova del Barranc del Migdia	Dia1	CBMX'10 UE 46 (41)	Beta-300 992<br>Beta-292 719<br>Beta-296 221<br>Beta-300 991	4070±30<br>4040±40<br>4020±30<br>3800±40	2851-2491 BCE<br>2839-2469 BCE<br>2619-2471 BCE<br>2451-2059 BCE	Adult female	Copper Age
Cova del Barranc del Migdia	Dia3	CBMX'10 UE 26 (56)	Beta-300 992<br>Beta-292 719<br>Beta-296 221<br>Beta-300 991	4070±30<br>4040±40<br>4020±30<br>3800±40	2851-2491 BCE<br>2839-2469 BCE<br>2619-2471 BCE<br>2451-2059 BCE	Adult male	Copper Age

References:

- Soler Díaz, J,A., Roca de Togores Muñoz, C., Esquembre Beviá, M.A., Gómez Pérez, O., Boronat Soler, J.D., Benito Iborra, M., Ferrer García, C. & Bolufer Marqués, J., 2016. Progresos en la investigación del fenómeno de inhumación múltiple en la Marina Alta (Alicante). A propósito de los trabajos desarrollados en la Cova del Randero de Pedreguer y en la Cova del Barranc del Migdia de Xàbia. *Del neolític a l'edat de bronce en el Mediterrani Occidental. Estudis en homenatge a Bernat Martí Oliver*. TV SIP 119. Museo de Prehistoria de Valencia, pp.323-348.

Cova del Cantal (Biar, Alicante, Spain)

Soler Díaz, J.A., Roca de Togores Muñoz, C. & Esquembre Beviá, M.A.

Burial cavity located 850 meters above sea level in the Sierra of Reconco. The "U" shape plan presents two inlets and three rooms, one of which *–room 3-* was blocked at the beginning of the excavation in 1990, when scattered human remains were discovered in all areas as well as an ossuary of over 20 cm thickness (subsector 5B). Here seven skulls were found along with 6 vessels, 2 copper awls, an axe and 12 arrowheads. It has been suggested that the cavity was a morgue, where the disarticulated remains were stacked up forming the ossuary. It should be noted that in previous uncontrolled interventions a fragment of Bell Beaker pottery was discovered along with other metallic elements such as a tongue dagger. Based on radiocarbon dates, it can be confirmed that that the funerary use took place in the 3rd millennium cal BCE and early 2nd millennium BCE.

SITE	SAMPLE NUMBER	ARCHAEOLOGICAL CONTEXT	LAB ID	DATE BP	CALIBRATED DATA 2σ	AGE/SEX	PERIOD
Cova del Cantal	Coca1	CC ´90 Caja 3 No 2	-	-	-	Child	Copper Age
Cova del Cantal	Coca2	CC'90 Caja 3 No 20c	Beta-422720	4020±30	2615-2470 BCE	Adult indet	Copper Age
Cova del Cantal	Coca3	CC'90 Caja 4 No 2	Beta-422721	3490±30	1890-1700 BCE	Adult indet	Late Copper Age/Bronze Age
Cova del Cantal	Coca4	CC'90 Caja 4 No 55	-	-	-	Adult indet	Copper Age
Cova del Cantal	Coca5	CC'90 Caja 3 No 16	-	-	-	Adult indet	Copper Age

References:
- López Seguí, E.J., García Bebia, M.A. & Ortega Pérez, J.R., 1990. La Cova del Cantal (Biar, Alicante). *Lucentum,* IX-X, pp. 25-49.

Cova d' En Pardo (Planes, Alicante, Spain)

Soler Díaz, J.A., Roca de Togores Muñoz, C.

This cavity opens about 680 m a.s.l. on the southern slope of the Serra de la Albureca. The floor level consists of two different areas separated by a large formation of stalagmites. The left area measures 11 by 6 meters in its largest part, while the roof lacks any stalactitic columns. The right area measures

14 by 8 meters and has a high vault with speleothems in the background. Excavations started in 1965 under Miquel Tarradell and continued from 1993 to 2007 by the MARQ of Alicante with a multidisciplinary approach. The actions overall reveal a powerful stratigraphic complex covering the periods from Upper Palaeolithic to the Bronze Age. Levels III and IIb are related to a Late Neolithic funerary site. The deposit from these areas contained human pertaining to remains thirty individuals dating from c. 3850-2850 cal BCE. No complete skeletons have been found. It is believed that human remains, once disarticulated, where placed against the walls and the bottom of the cavity, conforming an ossuary. While no metallic elements have been found, among the funerary offerings we find an interesting set of adornments and costume elements in bone, as well as pottery with no decoration and a lithic industry with very elaborate arrowheads.

SITE	SAMPLE NUMBE R	ARCHAEOLOGICAL CONTEXT	LAB ID	DATE BP	CALIBRATED DATA 2σ	AGE/SEX	PERIOD
Cova d'en Pardo	Par1	ENP'98 6.6 D III (48)	Beta-231 886<br>Beta 203 493<br>Beta-231 875	4430±40<br>4490±40<br>4550±40	3330-2922 BCE<br>3350-3030 BCE<br>3484-3100 BCE	Adult female	Late Neolithic
Cova d'en Pardo	Par2	ENP'97 5.6 A IIb (79)	Beta-95394	4270±50	3022-2680 BCE	Adult female	Late Neolithic
Cova d'en Pardo	Par3	ENP '98 6.6 D llb(15) /5.6A lIb				Adult female	Late Neolithic
Cova d'en Pardo	Par4	ENP' 99 5.4 DIII (17)	Beta- 231886<br>Beta-203 493<br>Beta-231 875	4430±40<br>4490±40<br>4550±40	3330-2922 BCE<br>3350-3030 BCE<br>3484-3100 BCE	Adult indet	Late Neolithic
Cova d'en Pardo	Par5	Enp'94 4.5 llb (49)/ENP'94 5.4 BIII				Adult male	Late Neolithic
Cova d'en Pardo	Par7	ENP ·95 7.5 AIII (25)				Child	Late Neolithic

References:
- Soler Díaz, J.A. & Roca de Togores Muñoz, C., 2012. Ritual funerario en la Cova d’En Pardo ca. 3.350-2.850 CAL ANE: espacialidad, cronología y territorio cultural. In J.A. Soler Díaz, Coord. *Cova d’en Pardo. Arqueología en la Memoria. Excavaciones de M. Tarradell, V. Pascual y E. Llobregat (1961-1965), catálogo de materiales del Museo de Alcoy y estudios a partir de las campañas del MARQ (1993-2007) en la cavidad de Planes, Alicante*. Fundación C.V. MARQ y Ayuntamiento de Alcoy, Alicante-Alcoy, pp. 205-248.

El Alto del Reinoso (Fresno de Rodilla/Monasterio de Rodilla, Burgos, Spain)

Rojo Guerra, M.A.

The site of Alto del Reinoso is a Neolithic collective grave located on top of a small hill, part of a narrow platform in the Paramo highlands, which dominates the entire surrounding area from a striking topographical location. It was excavated between 2006 and 2007 by archaeologists from the Valladolid University (Spain). Although this tomb lacks a megalithic structure, it is closely linked with this phenomenon by the features of the ossuary and grave goods. Most probably it was originally a collective sepulture made of vegetal materials (house of the dead), of around 3.5 m of diameter, that was eventually dismantled, closed off and finally turned into a monumental structure by erecting a stone mound above the ossuary. A minimum number of 47 individuals were identified including males, females, and subadults, although children aged 0-6 years were underrepresented. The bones were found on two layers, in the top one were secondary burials, and in the bottom complete primary burials in situ. Amongst the grave goods disposed with the dead were both personal adornments (stone necklace beads, wild boar tusk pendants) and tools (polished stone axes, flint blades and microliths, bone spatulas). Extensive DNA, Sr and C/N isotope research was carried out in the frame of this

project by a team from the Mainz University, however the data were published previously (Alt et al., 2016). Radiocarbon dates from human bones shown a chronology of the first half of 4th millennium BCE (MAMS-14327, 4933±27 BP, 3768-3653 cal BCE; MAMS-14326, 4854±26 BP, 3697-3540 cal BCE).

SITE	SAMPLE NUMBER	ARCHAEOLOGICAL CONTEXT	LAB ID	DATE BP	CALIBRATED DATA 2σ	AGE/SEX
El Reinoso	Rein1	Ind. 1	MAMS-14325	4911±25	3759-3644 BCE	Adult Female
El Reinoso	Rein2	Ind. 2	-	-	-	Adult Male
El Reinoso	Rein3	Ind. 3	-	-	-	Adult Male
El Reinoso	Rein4	Ind. 4	-	-	-	Adult Female
El Reinoso	Rein5	Ind. 5	-	-	-	Juvenile Male
El Reinoso	Rein6	Ind. 6	-	-	-	Adult ind
El Reinoso	Rein8	Ind. 8	-	-	-	Adult
El Reinoso	Rein9	Ind. 9	-	-	-	Adult Female
El Reinoso	Rein10	Ind. 10	-	-	-	Adult Female
El Reinoso	Rein12	Ind. 12	-	-	-	Adult male
El Reinoso	Rein13	near Inv. 1173-74	-	-	-	Child
El Reinoso	Rein14	Inv. 1171-72	-	-	-	Adult male
El Reinoso	Rein15	Inv. 1149-50	-	-	-	Adult
El Reinoso	Rein16	Inv. 1158-60	-	-	-	Adult male
El Reinoso	Rein17	Inv. 1022-23	MAMS-14326	4854±26	3697-3540 BCE	Juvenile
El Reinoso	Rein18	Inv. 3102-05	-	-	-	Juvenile Male
El Reinoso	Rein19	Inv. 2867-70	-	-	-	Adult male
El Reinoso	Rein20	Inv. 2835-38	-	-	-	Child
El Reinoso	Rein21	Inv. 1295-98	-	-	-	Adult
El Reinoso	Rein22	Inv. 2948-51	-	-	-	Juvenile
El Reinoso	Rein23	Inv. 1010-11	-	-	-	Juvenile
El Reinoso	Rein24	Inv. 2799-801	-	-	-	Juvenile
El Reinoso	Rein28	Inv. 1014-15	-	-	-	Adult

References:

- Alt, K.W. et al., 2016. A community in life and death: the Late Neolithic megalithic tomb at Alto de Reinoso (Burgos, Spain), *PLOS ONE 11(1),* e0146176. doi: 10.1371/journal.pone.0146176.
- Rojo Guerra, M. A. et al., 2015. El tiempo y los ritos de los antepasados. La Mina y el Alto del Reinoso, novedades sobre el megalitismo en la cuenca del Duero, *ARPI Arqueología y Prehistoria del Interior Peninsular*, Vol 03-2015, pp. 133-147.

El Hundido (Monasterio de Rodilla, Burgos, Spain)

Alonso Fernández, C. & Jiménez Echevarría, J.

El Hundido was excavated in 2005 by Cronos SC Arqueología y Patrimonio, under the direction of Carmen Alonso. Geographically it is located in the natural corridor that connects the Meseta and the high basin and half of the Valle del Ebro. The site is integrated by a collective burial of the Late Neolithic-Chalcolithic of nearly a hundred individuals. The first funerary ritual ended with the deliberated destruction of the monument by fire (Alonso, 2015). About 500 years after, the site had new funerary use within Bell Beaker context, with the construction of three individual graves made of stone (Alonso, 2013). One of the tombs studied was constructed taking advantage of the small corridor from the collective burial. In the tomb a man (older than 55 years), was buried in fetal position with one Ciempozuelos style Bell Beaker, one Palmela point, one bone point and one sphere of pyrite as grave goods. A radiocarbon date from a human bone, places the grave at the transition between Copper Age and Ancient Bronze Age. The other tombs were occupied by a man older than 45 years, accompanied with one Ciempozuelos style Beaker carinated bowl and one bi-pointed copper awl, and a 50-55-year-old man without funerary goods. The characteristics of the funerary goods provide important new knowledge of the cultural connections between the Valle del Duero and the Valle del Ebro.

SITE	SAMPLE NUMBER	ARCHAEOLOGICAL CONTEXT	LAB ID	DATE BP	CALIBRATED DATA 2σ	AGE/SEX	PERIOD
El Hundido	Hund1	UE 750	-	-	-	Adult male	Late Copper Age
El Hundido	Hund2	UE 450	CSIC-1896	3933±32	2490-2335 BCE	Adult male	Late Copper Age

References:
- Alonso, C., 2013. Las tumbas campaniformes del monumento funerario El Hundido (Monasterio de Rodilla, Burgos). *Munibe* 64, pp. 89-103.
- Alonso, C., 2015. La tumba colectiva de El Hundido (Monasterio de Rodilla, Burgos) y su ritual funerario durante el Neolítico Final y el Calcolítico. *Trabajos de Prehistoria* 72(1), pp. 84-104.

El Juncal (Getafe, Madrid, Spain)

López Jiménez, O., Martínez Calvo, V.

The site *El Juncal* was excavated in 2011 by GIPSIA, Ltd as part of a rescue archaeologic campaign in the northwestern area of Getafe (Madrid). Not far from the *Manzanares* river terraces, it oversees the surrounding plains from a gentle height. The main site occupation corresponds to what we know as “ditched enclosure”. At least two main ditches and three occupation areas were recorded during the digging of the more than 4.000 m^2 opened at that time. That was merely half of the entire area of the settlement. In the second and third areas there were recovered the evidences of two mass graves and four individual burials corresponding to Chalcolithic period (Del Olomo et al., 2014). Some of them are clearly related to bell-beaker pottery, like in the case of the maritime bell-beaker associated to burial 301. On the other hand, the collective burials lack any material deposits or grave goods. Those collective burials, located into big holes, excavated in the natural substrate were formed by two groups of 5 and 12 individuals respectively. The living areas yielded also many remarkable Chalcolithic items including phalanx idols, Palmela arrowheads, v-shaped buttons and evidences of

different types of Bell Beaker sets. Occupation will continue during early Bronze Age but showing a clear decreasing of population and associated structures.

SITE	SAMPLE NUMBER	ARCHAEOLOGICAL CONTEXT	AGE/SEX	LAB ID	DATE BP	CALIBRATED DATA 2σ	PERIOD
El Juncal	Junc1	UE 03 Est 93 Ind 6	Adult Male				Copper Age
El Juncal	Junc2	UE 03 Est 93 Ind 5	Adult Male				Copper Age
El Juncal	Junc3	UE 01 Est 199 Ind 2	Juvenile Female				Copper Age
El Juncal	Junc4	UE 03 Est 93 Ind 9	Ind				Copper Age
El Juncal	Junc5	UE 03 Est 93 Ind 4	Adult Female				Copper Age
El Juncal	Junc6	UE 01 Est 93 Ind 1	Adult Male				Copper Age
El Juncal	Junc7	UE 03 Est 93 Ind 8	Adult Female				Copper Age
El Juncal	Junc8	UE 01 Est 199 Ind 4	Adult Female	LTL12782A	4026 ± 40	2835-2467 BCE	Copper Age
El Juncal	Junc9	UE 01 Est 199 Ind 1	Adult Male				Copper Age
El Juncal	Junc10	UE 03 Est 93 Ind 3	Adult Female				Copper Age
El Juncal	Junc11	UE 03 Est 93 Ind 11	Adult Female				Copper Age
El Juncal	Junc12	UE 01 Est 199 Ind 5	Adult Ind				Copper Age
El Juncal	Junc13	UE 03 Est 93 Ind 7	Child				Copper Age
El Juncal	Junc14	UE 01 Est 199 Ind 3	Adult Male				Copper Age

References:
- Del Olmo Calvin, A. et al., 2014. Los enterramientos del yacimiento El Juncal (Getafe, Madrid). *Actas de las VIII jornadas de Patrimonio Arqueológico en la Comunidad de Madrid,* pp. 515.

Els Trocs Cave (San Feliu de Veri-Bisaurri, Huesca, Spain)

Rojo Guerra, M.A.

The site of Els Trocs was excavated during six seasons (2009-2012, 2014 and 2016) under the direction of Manuel A. Rojo Guerra (Valladolid University) and José I. Royo Guillén (Aragon Government), within the framework of the research project "Pathways of the Neolithic" (HAR2009-09027) (Rojo et al., 2012). The site is located near the axial Pyrenees, 1500 m high, near the headwaters of the Ésera and Isábena rivers. It is a cave with four occupation levels, three of them belong to the Neolithic period and another is dated in roman times. During the first occupation of the Early Neolithic (Mams-16163, 6285±25 BP, 5315-5215 cal BCE; Mams-16168, 6249±28 BP, 5310-5200 cal BCE –Rojo *et al.*, 2013-) different human bones with no anatomical connection (around seven individuals) and clear evidences of *peri mortem* manipulation were found on a floor made of thousands of potsherds paving the entire cave. The second phase has several radiocarbon dates on cereal samples (Beta-316511, 5590±40 BP, 4500-4340 cal BCE; Beta-316515, 5590±40 BP, 4500-4340 cal BCE; Beta-319513, 5580±40 BP, 4490-4340 cal BCE –Rojo *et al.*, 2013-), belonging to the Middle Neolithic. Another floor was discovered here, made of small stones. This occupation is mainly related with sheep and goat seasonal farming. The third phase can be attributed to the Middle/Late Neolithic by several radiocarbon dates made on human bone samples (Mams-16165, 5035±23 BP, 3945-3769 cal BCE; Mams-16160, 5008±23 BP, 3934-3709 cal BCE; Mams-14856, 5005±27 BP, 3820-3700 cal BCE; Mams-16167, 4512±25 BP, 3350-3101 cal BCE –Rojo *et al.*, 2013). Here also pastoralist and transhumance activities are documented, but also two pits with human bones in

secondary position. This site illustrates livestock specialization at the beginning of the Early Neolithic, related with the spring-summer quality pastures of this high mountain environment.

References:

SITE	SAMPLE NUMBER	ARCHAEOLOGICAL CONTEXT	LAB ID	DATE BP	CALIBRATED DATA 2σ	PERIOD
Els Trocs	Troc1	UE 63 C: 558 S:6 Nº Inv: 1623	Mams-16159	6280±25	5315-5215 BCE	Early Neolithic
Els Trocs	Troc2	UE 69 C: 589 S:7 Nº Inv: 14227	Mams-16160	5008±23	3934-3709 BCE	Middle/Late Neolithic
Els Trocs	Troc3	UE 82 C: 560 S:7 Nº Inv: 14296	Mams-16161	6217±25	5180-5060 BCE	Early Neolithic
Els Trocs	Troc4	UE 85 C: 557 S:9 Nº Inv: 14402	Mams-16162	6218±24	5180-5060 BCE	Early Neolithic
Els Trocs	Troc5	UE 101 C: 558 S:5 Nº Inv: 22743-22745	Mams-16164	6249±25	5310-5200 BCE	Early Neolithic
Els Trocs	Troc6	UE:1 C: 650 S:1 Nº Inv: 22404	Mams- 16165	5035±23	3945-3769 BCE	Middle/Late Neolithic
Els Trocs	Troc7	UE 20 C: 617 S:3 Nº Inv: 22658	Mams 16166	6234±28	5310-5200 BCE	Early Neolithic
Els Trocs	Troc8	UE:6 2009:13	Mams- 16167	4512±25	3350-3101 BCE	Late Neolithic
Els Trocs	Troc10	UE 20 C: 587 S: 9 Nº Inv: 22481	Mams 16168	6249±28	5310-5200 BCE	Early Neolithic
Els Trocs	Troc12	UE 98 C:618 S9 No Inv:22722	Mams 14856	5005±27	3820-3700 BCE	Middle/Late Neolithic
Els Trocs	Troc13	UE 105 C: 619 S: 8 Nº Inv: 23017-18	Mams 16163	6285±25	5315-5215 BCE	Early Neolithic

- Rojo, M. et al., 2012. Los caminos del neolítico: Un proyecto de investigación en el valle del Ebro. Actes. Xarxes al Neolitic. Congrés Internacional. *Rubricatum* 5. Revista Del Museu de Gavá. Barcelona, pp. 43-50.
- Rojo, M. et al., 2013. Pastores trashumantes del neolítico antiguo en un entorno de alta montaña: Secuencia crono-cultural de la cova de Els Trocs (San Feliú de Veri, Huesca). *BSSS arqueología* LXXIX. Boletín del Seminario de Estudios de Arte y Arqueología. Universidad de Valladolid, pp. 9-55.
- Rojo, M. et al., 2014. Los primeros pastores trashumantes de al Alta Ribagorza. In I. Clemente, E. Gassiot & J. Rey, eds. *Sobrarbe antes de Sobrarbe. Pinceladas de historia de los Pirineos*. Centro de Estudios de Sobrarbe. Comarca de Sobrarbe, pp. 127-151.
- Rojo, M. et al., 2015. La Cueva de Els Trocs: Un asentamiento del Neolítico Antigo junto al Pirineo Axial. In V. Gonçalves, M. Diniz, & A.C.Sousa, eds. *V Congreso do Neolitico Peninsular, Estudios y Memorias*, 8. Centro de Arqueologia da Universidade de Lisboa. UNIARQ WAPS, pp. 189-197.

Es Forat de Ses Aritges (Menorca, Spain)

Lull, V., Micó, R., Rihuete-Herrada, C. & Risch, R.

Es Forat is collective burial site located in the southwestern part of the island of Menorca. It was excavated by the Universitat Autònoma de Barcelona in 1999 (Lull et al., 1999, 2006). Like the well-known Cova des Càrritx, located only four meters further north, this natural cave was sealed off with a cyclopean stone wall. A narrow opening allowed to access the funerary space of c. 14 m^2, in which about 100 individuals had been interred over several centuries. According to the nine available 14C dates obtained from different, randomly sampled individuals the funerary structure was used between 1400-1000 cal BCE (KIA-44842r, 2730±30 BP, 928-813 cal BCE (de Cet, 2014) KIK-962/UtC-7856, 3170±35, 1509-1321 cal BCE (Lull et al., 1999)). Thousands of human bones were recovered, but few were still in anatomical connection. Together with these remains, a variety of grave offering

appeared, mainly pottery, copper and tin artefacts (bracelets, awls, beads) and objects made out of bone or teeth (V-perforated buttons, pendants, awls, etc.).

SITE	SAMPLE NUMBER	ARCHAEOLOGICAL CONTEXT	PERIOD
Es Forat de Ses Aritges	Fora1	FA 32-3592	Late Bronze Age
Es Forat de Ses Aritges	Fora2	FA 47-8890	Late Bronze Age
Es Forat de Ses Aritges	Fora3	FA 47-12159	Late Bronze Age
Es Forat de Ses Aritges	Fora4	FA 49-9819	Late Bronze Age

References:
- De Cet, M., 2014. Long-term social development on a Mediterranean Island: Menorca between 1600 BCE and 1900 CE. PhD Thesis at the University of Kiel, Kiel (in press).
- Lull, V. et al. 1999. La Cova des Cárritx y la Cova des Mussol. Ideología y Sociedad en la Prehistoria de Menorca. Barcelona. Consell Insular de Menorca.
- Lull, V. et al., 2006. Los botones con perforación en "v" de es Forat de ses Aritges (Ciutadella, Menorca): implicaciones económicas y rituales, Revista de Menorca, Maó. In N. Ferrera Bicho, ed. Animais na pré-historia e Arqueologia da Península Ibérica – Actas do IV Congreso de Arqueologia Peninsular, Centro de Estudos de Património, Universidade do Algarve, pp. 57-67.

Fuente Álamo (Cuevas del Almanzora, Almería, Spain)

Risch, R.

Fuente Álamo is a typical El Argar hilltop settlement located at the southern fringes of the Almagro mountain range. After the first explorations at the end of the 19th century (Siret & Siret 1887), between 1977-1996 the German Archaeological Institute excavated ca. 1500 m^2 and 64 burials in this 2 ha large site (Schubart et al., 2001; Risch, 2002; Schuhmacher & Schubart, 2003, Schubart, 2012). The stratigraphic sequence reveals a continuous occupation from the beginning of El Argar until the Late Bronze Age (ca. 2200-1400 cal BCE). This sequence can be divided into four settlement horizons according to the successive constructive phases. At the height of its development, between ca. 1900-1600 cal BCE, the settlement was organized into at least three sectors. The most monumental buildings and the richest individual or double tombs were located on the summit and the upper eastern slope. Here, high quality grinding stones, most of the forging and metal sharpening tools, as well as over 90% weight of the metal deposited in intramural burials have been found. The western slope presents the largest number of burials and appears to have been densely settled. Finally, the southern slope was an important productive sector. Specialised cereal processing workshops and grinding stone storage rooms were placed on successive terraces. Based on the number of grinding stones found in the settlement it has been calculated that over 2000 persons could have supplied with flour (Risch, 2002). During the middle and late El Argar period, Fuente Álamo seems to have represented a regional center for cereal storage, processing and redistribution.

SITE	SAMPLE NUMBER	ARCHAEOLOGICAL CONTEXT	LAB ID	DATE BP	CALIBRATED DATA 2σ	AGE/SEX	PERIOD
Fuente Álamo	Fala1	75	OxA-4972	3545 ± 65	2114-1693 BCE	Adult female	Early Bronze Age
Fuente Álamo	Fala2	75a	OxA-4973	3635 ± 50	2187-1884 BCE	Adult male	Early Bronze Age
Fuente Álamo	Fala5	112	-	-	-	Adult female	Early Bronze Age

References:
- Hedges, R.E.M. et al., 1995. Radiocarbon dates from the Oxford AMS System: Archaeometry Datelist 19. *Archaeometry* 37 (1), pp. 195-214.
- Risch, R., 2002. *Recursos Naturales, Medios de Producción y Explotación Social. Un Análisis Económico de la Industria Lítica de Fuente Álamo (Almería), 2250-1400 Antes de Nuestra Era*, Iberia Archaeologica, 3. Philipp von Zabern, Mainz.
- Schubart, H., 2012. *Die Gräber von Fuente Álamo. Ein Beitrag zu den Grabriten und zur Chronologie der El Argar-Kultur*. Madrider Beiträge 32, Deutsches Archäologisches Institut Madrid-Reichert, Wiesbaden.
- Schuhmacher, T. X. & Schubart, H., 2003. *Fuente Álamo, Die Siedlungskeramik der Grabungen 1985–1991. Stratigraphisch geordnete Keramik der el Argar-Zeit aus den Grabungen 1977-1982.* Iberia Archaeologica, 4, Philipp von Zabern, Mainz.
- Schubart, H., Pingel, V., & Arteaga, O. (eds), 2001. Fuente Álamo, Teil 1: Grabungen von 1977 bis 1991 in einer bronzezeitlichen Höhensiedlung Andalusiens, Madrider Beiträge, 25, Philipp von Zabern, Mainz.
- Siret, H. & Siret, L., 1887. *Les Premières Âges du Métal dans le Sud-est de l'Espagne*. Antwerpen.

Fuente Celada (Quintadueñas, Burgos, Spain)

Rojo Guerra, M.A.

Fuente Celada is a settlement with many rock-cut pits located in Quintanadueñas. The site was excavated in 2008 (Alameda Cuenca-Romero et al., 2011), and was partially occupied during the Neolithic, but mainly during the Early Copper Age. Together with indications of a domestic area ("silos", post holes, mud fragments used in the huts, stone mortars and other domestic remains), a few human graves were discovered inside several pits. Pit 62 had an old woman in crouched position with three bone rings around the neck, which was dated to the Early Neolithic (UGA-7565). The second grave is located on Pit 19, where a young woman was disposed in an unusual inverted position (almost vertically placed, with the head at the bottom and the feet at the top). Interestingly, this pit shows the most important accumulation of potsherds in the site (1018, 19.5% of the total). This grave has been dated in the Copper Age (UGA-7562). Finally, in Pit 5 partial and disconnected remains of three individuals (a subadult and two adult men) were documented, also belonging to the Copper Age (UGA-7560).

SITE	SAMPLE NUMBER	ARCHAEOLOGICAL CONTEXT	LAB ID	DATE BP	CALIBRATED DATA 2σ	AGE/SEX	PERIOD
Fuente Celada	Cel3	H62 UE 622	UGA-7565	6120±30	5207-4961 BCE	Adult female	Early Neolithic
Fuente Celada	Cel2	H19 UE 193	UGA-7562	4100±25	2859-2574 BCE	Adult female	Early Copper Age
Fuente Celada	Cel1	H5 UE 53 Ind 3	UGA-7560	4030±25	2619-2475 BCE	-	Early Copper Age

References:

- Alameda Cuenca-Romero, M.C. et al., 2011. El ‘campo de hoyos’ calcolítico de Fuente Celada (Burgos): datos preliminares y perspectivas. Complutum 22(1), pp. 47–69.

Fuentepecina I (Sedano, Burgos, Spain)

Rojo Guerra, M.A.

This megalithic grave was excavated by the Valladolid University in a cemetery where 5 tombs were discovered, although just 4 have been studied. The stone mound is located on top of a natural elevation over the surrounding landscape. It is a small passage grave severely altered by remotions from historical times. Only four orthostats of the small chamber (around 2.5 m of diameter) and two in the beginning of the corridor (oriented to the south east) remained intact. Abundant disarticulated human bones were recovered from the original Neolithic burial layer. Grave goods were scarce though, with several flint microliths, a single necklace bead, and two splendid decorated bone idol-spatulas. Outside the chamber a charcoal sample was tested which resulted in the following radiocarbon date: GrN. 16073 270±140 BP (4366-3721 cal BCE) (Delibes & Rojo Guerra, 1997), perhaps belonging to the erection of the megalithic tomb.

SITE	SAMPLE NUMBER	ARCHAEOLOGICAL CONTEXT	PERIOD
Fuentepecina I	Fupe6	Quadr NE	Midlle/Late Neolithic
Fuentepecina I	Fupe7	Quadr Central 50x50	Midlle/Late Neolithic
Fuentepecina I	Fupe8	Quadr. SE 2x2	Midlle/Late Neolithic
Fuentepecina I	Fupe9	Quadr S 1x1	Midlle/Late Neolithic

References:
- Delibes, G. & Rojo Guerra, M.A., 1997. C-14 y secuencia megalítica en La Lora burgalesa: acotaciones a la problematica de las dataciones absolutas referentes a yacimientos dolmenicos. In A Rodríguez Casal ed. *O Neolítico Atlantico e as Orixes do Megalitismo*. Santiago de Compostela: Xunta de Galicia, pp. 391 -414.

Fuentepecina II (Sedano, Burgos, Spain)

Rojo Guerra, M.A.

This megalithic grave was excavated on September of 1990 by a team from the Valladolid University. It is a simple dolmen with a small pentagonal stone chamber surrounded by a stone oval mound of 17.5 x 12 m of diameter and 1.5 m of height. Inside this megalithic chamber an ossuary was discovered where bones were disposed in secondary position. Skulls were especially well preserved compared with the rest of the bones. No bioanthropological analyses had been made on these bones, but at least by the number of complete skulls a minimum number of 18 individuals could be estimated. Abundant grave goods were recovered with the human bones, especially hundreds of stone necklace slate beads, 50 flint microliths, blades, and several bone spatulas. Three ophite stone polished axes were discovered at the bottom of the tomb, as a sort of foundation ritual. There are two Radiocarbon dates: GrN-18669, 5375±45 BP, 4333-4056 cal BCE; GrN-18667, 5170±100 BP, 4239-3714 cal BCE.

SITE	SAMPLE NUMBER	ARCHAEOLOGICAL CONTEXT	PERIOD
Fuentepecina II	Fupe1	FP2-M2	Midlle/Late Neolithic
Fuentepecina II	Fupe3	FP2-M1	Midlle/Late Neolithic
Fuentepecina II	Fupe4	FP2-M5	Midlle/Late Neolithic
Fuentepecina II	Fupe5	FP2-M7	Midlle/Late Neolithic

References:
- Delibes, G. & Rojo Guerra, M.A., 1997. C-14 y secuencia megalítica en La Lora burgalesa: acotaciones a la problematica de las dataciones absolutas referentes a yacimientos dolménicos. In A Rodríguez Casal ed. *O Neolítico Atlantico e as Orixes do Megalitismo*. Santiago de Compostela: Xunta de Galicia, pp. 391 -414.

Fuentepecina IV (Sedano, Burgos, Spain)

Rojo Guerra, M.A.

This was the last megalithic sepulture excavated by the Valladolid University in this Neolithic cemetery in 1990. It is a small mound of 10 m of diameter and just 0.5 m high, which was only visible because it was located on top of a natural elevation. Inside the chamber but really close to the surface removed disarticulated human bones were discovered, accompanied by extremely rich grave offerings: around 50 flint microliths, 50 flint blades, cores, thousands of slate necklace and dentalium type beads, quartz prisms, and ten stone polished axes, etc. There are no radiocarbon dates available but the grave goods and the tomb structure are typical from the megalithism in the Northern Meseta.

SITE	SAMPLE NUMBER	ARCHAEOLOGICAL CONTEXT	PERIOD
Fuentepecina IV	Fupe10	no name (disarticulated human bones)	Midlle/Late Neolithic
Fuentepecina IV	Fupe11	no name (disarticulated human bones)	Midlle/Late Neolithic

References:
- Delibes De Castro, G., Rojo-Guerra, M. A. & Represa Bermejo, J.I., 1993. Dólmenes de la Lora, Burgos. Guía arqueológica, Junta de Castilla y León, Consejería de Cultura y Turismo, pp. 74-81.

Gruta da Malgasta (Peniche, Portugal)

Cardoso, J.L. & Kunst, M.

This burial cave, formed in Jurassic limestone, near the Lapa Furada and Casa da Moura caves, was explored by Joaquin Filipe Nery Delgado at the same time as the thow other caves (Delgado, 1867). The grave goods that are kept in the Museu do Laboratório Nacional de Energia e Geologia were published in 1992 (Carreira & Cardoso, 1992) with some of the objects from Lapa Furada, due to the fact that some fragments from the same vessel were attributed to both caves. The most important pieces have labels from that time with the name of Lapa Furada, some of them were published by E. Cartailhac (Cartailhac, 1886, Fig. 118). Material typologies from the published pieces suggest successive phases of use of the burial cave ranging from the Late Neolithic to the Bronze Age.

SITE	SAMPLE NUMBER	ARCHAEOLOGICAL CONTEXT	PERIOD
Gruta da Malgasta	Malga1	1	Late Neolithic-Bronce Age
Gruta da Malgasta	Malga2	2	Late Neolithic-Bronce Age

References:
- Carreira, J. R. & Cardoso, J. L., 1992. Escavações de Nery Delgado no planalto de Cesareda nas grutas da Lapa Furada e da Malgasta (Peniche): estudo do espólio arqueológico. Com*unicações dos Serviços Geológicos de Portugal*. Lisboa. 78 (2), pp. 145-173.
- Cartailhac, É.,1886. *Les Ages préhistoriques de L´Espagne et du Portugal*. Paris: Ch. Reinwald.
- Delgado, J. F. N, 1867. *Notica acerca das grutas da Cesareda*. Lisboa: Typographia da Academia Real das Sciencias.

Gruta das Alcobertas (Rio Maior, Portugal)

Cardoso, J.L. & Kunst, M.

Burial grave in Jurassic limestone that was explored by the Secção dos Trabalhos Geológicos de Portugal at the same time as the Gruta do Carvahlal de Turquel, before the 1880 Congress. The grave goods that were partially inventoried at that time (Santos, Zbyszewski & Ferreira, 1971) are being studied by one of us (J.L. Cardoso). The cave was used as a cemetery during a short period of time, given that the archaeological materials belong just to the Middle Neolithic, a period still poorly known but with other burial sites recently published, such as the cemetery of Gruta do Lugar do Canto, Alcanena (Cardoso & Carvalho, 2009), the cave of Casa da Moura, Óbidos (Carvalho & Cardoso, 2010/2011), or Algar do Bom Santo, Cadaval (Carvalho, ed., 2014).

SITE	SAMPLE NUMBER	ARCHAEOLOGICAL CONTEXT	PERIOD
Guta das Alcobertas	Alco1	221127	Middle-Late Neolithic
Guta das Alcobertas	Alco2	221132	Middle-Late Neolithic

References:
- Santos, M. C., Zbyszewski, G. & Ferreira, O., 1971. A gruta pré-histórica das Alcobertas. 2.º Congresso Nacional de Arqueologia (Coimbra, 1970). Actas. Coimbra: Junta Nacional da Educação, 1, p. 97-106.
- Cardoso, J. L. & Carvalho, A. F. (2008) – A gruta do Lugar do Canto (Alcanede) e sua importância no faseamento do Neolítico do território português. Homenagem a Octávio da
-Carvalho, A. F. & Cardoso, J. L. (2010/2011) – A cronologia absoluta das ocupações funerárias da gruta da Casa da Moura (Óbidos). Estudos Arqueológicos de Oeiras. Oeiras. 18, p. 393-406.
-Carvalho, A. F., ed. (2014) – Bom Santo Cave (Lisbon) and the Middle Neolithic Scieties of Southern Portuga. Faro: Universidade do Algarve (Promontoria Monográfica 17).

Gruta de Nossa Senhora das Lapas (Tomar, Portugal)

Tomé, T.

Gruta de Nossa Senhora das Lapas was excavated between 1988 and 1994 by Luiz Oosterbeek and Ana Cruz. It corresponds to a small limestone cave, where different burial episodes were identified. Layer B included the commingled remains of several individuals (osteological analysis suggests a MNI of nine individuals in this layer). Both the material culture and radiocarbon dates indicate two different moments of burial occurring inside this layer, an earlier episode dating from the Early Neolithic (ICEN-805, 6100±70 BP, 5217-4841 cal BCE) and a later episode placed in the Middle Neolithic (I-17247, 5130±140 BP, 4258-3650 cal BCE) (Oosterbeek, 1993; 2003; Cruz, 1997). Osteological assessment was unable to identify clearly, which remains belong to each phase inside Layer B (Tomé, 2011; Tomé & Oosterbeek, 2011). A later burial was found in Layer A, delimited by a stone structure, corresponding to a male adult in a flexed position. Although no radiocarbon dates are available for this individual, the contents of the funerary package accompanying this burial (one ceramic pot and a metal axe) indicates that it must have been a Bell Beaker burial (Oosterbeek, 1993; 2003; Cruz, 1997).

SITE	SAMPLE NUMBER	ARCHAEOLOGICAL CONTEXT	PERIOD
Gruta de Nossa Senhora das Lapas	GSL1	GSL Q-Ind	Early/Middle Neolithic
Gruta de Nossa Senhora das Lapas	GSL2	GSL P25 S-C47	Early/Middle Neolithic
Gruta de Nossa Senhora das Lapas	GSL3	GSL LSS-C15	Early/Middle Neolithic

References:
- Cruz, A. R., 1997. *Vale do Nabão: do Neolítico à Idade do Bronze*. Arkeos 3. Tomar: CEIPHAR.
- Oosterbeek, L., 1993. Nossa Senhora das Lapas: Excavation of prehistoric cave burials in central Portugal. *Papers from the Institute of Archaeology*. 4, pp. 49-62.
- Oosterbeek, L., (2003). Megaliths in Portugal: the western network revisited. *In* T.G. Burenhult ed., *Stones and Bones. Formal disposal of the dead in Atlantic Europe during the Mesolithic-Neolithic interface 6000-3000 BC*. British Archaeological Reports – International Series. Oxford. 1201, pp. 27-37.
- Tomé, T., 2011. *Até que a Morte nos Reúna: Transição para o Agro-Pastoralismo na Bacia do Tejo e Sudoeste Peninsular*. PhD Dissertation. Vila Real: Universidade de Trás-os-Montes e Alto Douro.
- Tomé, T. & Oosterbeek, L., 2011. One Region, Two Systems? A paleobiological reading of cultural continuity over the agro-pastoralist transition in the North Ribatejo. In P. Bueno, E. Cerrillo & A. Gonzalez, Eds, *From the Origins: The Prehistory of the Inner Tagus Region*. Oxford: Archaeopress, pp. 43-54

Gruta do Cadaval (Tomar, Portugal)

Tomé, T.

Gruta do Cadaval is a limestone cave comprising two different rooms, excavated between 1983 and 1987 by Luiz Oosterbeek and Ana Cruz. Each room contained an individual burial, as well as commingled remains from several individuals. Human remains were extremely fragmented, precluding an accurate identification of individuals. Individual burials were radiocarbon dated to the Middle Neolithic (ICEN-803, 5390±50 BP, 4341-4056 cal BCE and ICEN-464, 5160±50 BP, 4143-3798 cal BCE) and the collective burial shows a chronology spanning from the Middle to the Late Neolithic (Beta-189995, 4640±40 BP, 3618-3350 cal BCE and I-17241, 5180±140 BP, 4326-3702 cal BCE) (Oosterbeek, 1994; 2003; Cruz, 1997). The collective deposition showed no signs of spatial organization, probably as a result of the depositions taking place quite close to the surface and of

trampling processes resulting from a recent use of this cave as a stable. A total MNI of 31 individuals was estimated for Gruta do Cadaval (Oosterbeek, 1994; Tomé, 2011; Tomé & Oosterbeek, 2011).

SITE	SAMPLE NUMBER	ARCHAEOLOGICAL CONTEXT	PERIOD
Gruta do Cadaval	CDV1	CDV SUP S-C39	Middle/Late Neolithic
Gruta do Cadaval	CDV2	CDV F30 S-C39	Middle/Late Neolithic
Gruta do Cadaval	CDV3	CDV H3 111	Middle/Late Neolithic

References:
- Cruz, A. R., 1997. *Vale do Nabão: do Neolítico à Idade do Bronze*. Arkeos 3. Tomar: CEIPHAR.
- Oosterbeek, L., 1994. *Echoes from the East: the western network. An insight to unequal and combined development, 7000-2000 BC*. PhD Dissertation. London: University College.
- Oosterbeek, L., 2003. Megaliths in Portugal: the western network revisited. In G. Burenhult, ed. *Stones and Bones. Formal disposal of the dead in Atlantic Europe during the Mesolithic-Neolithic interface 6000-3000 B.C.* British Archaeological Reports – International Series. Oxford. 1201, pp. 27-37.
- Tomé, T., 2011. *Até que a Morte nos Reúna: Transição para o Agro-Pastoralismo na Bacia do Tejo e Sudoeste Peninsular*. PhD Dissertation. Vila Real: Universidade de Trás-os-Montes e Alto Douro.
- Tomé, T. & Oosterbeek, L., 2011. One Region, Two Systems? A paleobiological reading of cultural continuity over the agro-pastoralist transition in the North Ribatejo. In P. Bueno, E. Cerrillo & A. Gonzalez, Eds, *From the Origins: The Prehistory of the Inner Tagus Region*. Oxford: Archaeopress, pp. 43-54

Gruta do Carvalhal de Turquel (Leiria, Portugal)

Cardoso, J.L. & Kunst, M.

It is a burial cave on jurassic limestones near Turquel (700 m) in the western coast and near the top of the Cabeço de Turquel. It was explored by the Secção dos Trabalhos Geológicos de Portugal, under the direction of Carlos Ribeiro and Nery Delgado, just before the IXth Session of the "Congresso Internacional de Antropologia e de Arqueologia Pré-Históricas" held at Lisbon in 1880, looking for archaeological materials for the Museu da Secção dos Trabalhos Geológicos de Portugal, which were finally published in 1974 (Spindler & Ferreira, 1974), excepting a small ritual vessel in the form of a pig that was published by E. Cartailhac (Cartailhac, 1886, Figs. 142-144) just after the mentioned conference. Given the tipology of the materials published and kept in the Museu do Laboratório Nacional de Energia e Geologia, this cave was used as a cemetery from the Early Neolithic, to which several decorated potsherds belong, amongst others (Spindler & Ferreira, 1974, Abb. 28), that were previously attributed to the Bronze Age (Spindler & Ferreira, 1974, Abb. 17, 129 a 132, Abb. 18, 134, 135, 140; Abb20, 153), but also during the Copper Age, including a Bell Beaker carinated bowl with geometric comb decoration, and finally also in the Bronze Age, because several typical carinated "taças" were discovered. The grave goods belonging to the Late Neolithic are also particularly important.

SITE	SAMPLE NUMBER	PERIOD
Gruta do Carvalhal de Turquel	Turq1	Copper/Bronze Age
Gruta do Carvalhal de Turquel	Turq2	Copper/Bronze Age

References:
- Cartailhac, É.,1886. *Les Ages préhistoriques de L´Espagne et du Portugal*. Paris: Ch. Reinwald.
- Spindler, K. & Ferreira, O. da Veiga., 1974. Das vorgeschichtliche fundmaterial aus der Gruta do Carvalhal/Portugal. *Madrider Mitteilungen*. Heidelberg. 15, p. 28-76.

Gruta dos Ossos (Tomar, Portugal)

Tomé, T.

Gruta dos Ossos was excavated from 1986 to 1989 by Luiz Oosterbeek and Ana Cruz. Funerary depositions inside this cave resulted in the formation of a deposit of the commingled remains of several individuals, with absolute dates and material culture indicating a chronology encompassing the Late Neolithic/Chalcolithic transition (Beta-189996, 4330±40 BP, 3083-2887 cal BCE; ICEN-465, 4630 ± 80 BP, 3633-3103 cal BCE and I-17368, 4460 ±110 BP, 3497-2889 cal BCE) (Oosterbeek, 1993; Cruz, 1997; Tomé, 2011; Tomé & Oosterbeek, 2011). A later radiocarbon date, obtained from bones recovered from layer IV (I-17248, 3970±140 BP, 2883-2060 cal BCE), suggests that funerary depositions were performed inside this cave up until the late 2nd millennium BCE. Nevertheless, the large margin of error associated with this radiocarbon sample suggests care must be taken in its interpretation. Its isolated nature – with all the remaining samples resulting in a consistent interval – strengthens the interpretation of Gruta dos Ossos as a Late Neolithic/Chalcolithic collective burial site. Osteological assessment indicates an MNI of 31 individuals (Tomé, 2011). Spatial dispersion of the human remains suggested a particular form of bone organization inside the deposit, where small piles of bones were constituted, with hip bones forming the base of such accumulations, followed by long bones, the crania remaining on top of such accumulations. An alignment of small limestone blocks near the entrance to the cave seemed to serve as a delimiting element for the burial zone. Material culture was apparently also spatially segregated, concentrating mostly outside the funerary deposition area (Oosterbeek, 1993).

SITE	SAMPLE NUMBER	ARCHAEOLOGICAL CONTEXT	PERIOD
Gruta dos Ossos	Gro2	GRO 9 VII 107	Late Neolithic/Copper Age
Gruta dos Ossos	Gro3	GRO 9 VI 44 + 195	Late Neolithic/Copper Age
Gruta dos Ossos	Gro4	GRO 9VI 10	Late Neolithic/Copper Age
Gruta dos Ossos	Gro5	GRO LGG S-C 27	Late Neolithic/Copper Age

References:
- Cruz, A. R., 1997. *Vale do Nabão: do Neolítico à Idade do Bronze*. Arkeos 3. Tomar: CEIPHAR.
- Oosterbeek, L., 1993. Gruta dos Ossos (Tomar) Um ossuário do Neolítico final. *Boletim Cultural de Tomar*. 18, pp. 11-28.
- Oosterbeek, L., 2003. Megaliths in Portugal: the western network revisited. In G. Burenhult, ed. *Stones and Bones. Formal disposal of the dead in Atlantic Europe during the Mesolithic-Neolithic interface 6000-3000 b.C.* British Archaeological Reports – International Series. Oxford. 1201, pp. 27-37.
- Tomé, T., 2006. *Reflexos da Vida na Morte: Paleobiologia das populações do Neolítico Final/Calcolítico do Vale do Nabão – Gruta dos Ossos*. Master Dissertation. Vila Real: Universidade de Trás-os-Montes e Alto Douro.
- Tomé, T., 2011. *Até que a Morte nos Reúna: Transição para o Agro-Pastoralismo na Bacia do Tejo e Sudoeste Peninsular*. PhD Dissertation. Vila Real: Universidade de Trás-os-Montes e Alto Douro.

- Tomé, T. & Oosterbeek, L., 2011. One Region, Two Systems? A paleobiological reading of cultural continuity over the agro-pastoralist transition in the North Ribatejo. In P. Bueno, E. Cerrillo & A. Gonzalez, Eds, *From the Origins: The Prehistory of the Inner Tagus Region*. Oxford: Archaeopress, pp. 43-54

Humanejos (Parla, Madrid, Spain)

Garrido-Pena, R., Flores-Fernández, R. & Herrero-Corral, A.M.

The site of Humanejos was excavated by Raúl Flores Fernández (PAGADEL) in several seasons between 2008 and 2011, as a rescue excavation. It is an extensive prehistoric habitat (more than 20 hectares), mostly with Copper Age occupation, on a soft slope close to the Humanejos stream where more than 2400 features (mostly pits) were discovered. Of them 1700 were Chalcolithic, mainly domestic, with the presence of a small enclosure (0.12 hectares of surface), but with more than 80 tombs also. The sepultures are extremely diverse, with both pre-Beaker and Bell Beaker ones (also not Beaker but contemporary with this phenomenon), and both with individual and multiple inhumations. Nine Bell Beaker tombs were excavated with extremely rich grave goods (decorated pottery, copper weapons as dagger, Palmela type point and even an axe and an Atlantic halbeard, gold jewelry, ivory V-perforated buttons and necklace beads, and stone wristguards). Pre-Beaker and not-Beaker graves occasionally have also important offerings as copper objects (daggers, awls) and adornments (stone necklace beads) (Flores & Garrido, 2012; 2014).

SITE	SAMPLE NUMBER	ARCHAEOLOGICAL CONTEXT	LAB ID	DATE BP	CALIBRATED DATA 2σ	AGE/SEX	PERIOD
Humanejos	Hume1	UE 443	-	-	-	Child	Copper Age
Humanejos	Hume2	UE 1461.87	Ua-40218	4009±56	2852-2346 BCE	Child	Early Copper Age
Humanejos	Hume3	UE 1461.54				Child	Early Copper Age
Humanejos	Hume4	UE 1461.43				Adult female	Early Copper Age
Humanejos	Hume8	UE 1461.76				Child	Early Copper Age
Humanejos	Hume19	UE 1461.2 Ind1				Adult male	Early Copper Age
Humanejos	Hume5	UE 455.2	Ua-40217<br>Ua-40218	3781±36<br>3825±37	2338-2046 BCE<br>2456-2146 BCE	Adult male	Late Copper Age
Humanejos	Hume10	UE 455.2				Adult male	Late Copper Age
Humanejos	Hume11	UE 455.9				Adult male	Late Copper Age
Humánenos	Hume12	UE 1853.4	Ua-43524	3917±33	2482-2295 BCE	Adult male	Late Copper Age
Humanejos	Hume14	UE 1853.6	Ua-43525	3797±32	2345-2136 BCE	Adult female	Late Copper Age
Humanejos	Hume15	UE 2014.1	Ua-43531	3907±33	2474-2294 BCE	Adult male	Late Copper Age
Humanejos	Hume18	UE 2014.42				Adult male	Late Copper Age
Humanejos	Hume21	UE 1964	-	-	-	Adult female	Late Copper Age
Humanejos	Hume24	UE 1750.2	-	-	-	Adult female	Bronze Age?
Humanejos	Hume25	UE 278.4	-	-	-	Juvenile	Copper Age

References:

- Flores-Fernández, R, & Garrido-Pena, R., 2014. Campaniforme y conflicto social: evidencias del yacimiento de Humanejos (Parla). In *IX Jornadas de Patrimonio Arqueológico en la Comunidad de Madrid* (2012, Alcalá de Henares, Madrid, España). Comunidad de Madrid (Dir. Gral. Patrim. Hist.), Sección Arqueología Col. Doctores y Licenciados, pp. 159-167.
- Flores-Fernández, R. & Garrido-Pena, R. (2012): "Excavaciones arqueológicas en el yacimiento de Humanejos (Parla, Madrid)". In VI Jornadas de Patrimonio Arqueológico en la Comunidad de Madrid (2009, Alcalá de Henares, Madrid, España). Comunidad de Madrid: 481-484.

- Liesau, C. et al., 2015. La mujer en el registro funerario campaniforme y su reconocimiento social. *Trabajos de Prehistoria*, 72(1), pp. 105-125.
- Ríos Mendoza, P., 2011. III. Nuevas fechas para el Calcolítico de la región de Madrid. Aproximación cronocultural a los primeros poblados estables. In C. Blasco, C. Liesau & P. Ríos eds. *Yacimientos calcolíticos con campaniforme de la región de Madrid: nuevos estudios*. Madrid. Manero Digital SL, pp. 73-86.
- Ríos Mendoza, P., 2013. New dating of the Bell Beaker Horizon in the region of Madrid. In M.P. Prieto & L. Salanova, coords. *Current researches on Bell Beakers, Proceedings of the 15th International Bell Beaker Conference: From Atlantic to Ural* (5th-9th May 2011, Poio, Pontevedra, Galicia, Spain). Santiago de Compostela, pp. 97-109.

Illeta dels Banyets (El Campello, Alicante, Spain)

López Padilla, J. A. & Soler Díaz, J.A.

This coastal settlement is located about 9 km northeast of the city of Alicante. At an uncertain time ago the original peninsula was torn apart from the coast due to an earthquake. In 1943, in order to link the little island again to the coast, a large part of the archaeological site was destroyed using dynamite. Currently, the estimated extension covers c. 0.4 ha, although it might have reached over one ha in the Bronze Age. The first excavations date back to the early twentieth century. Systematic excavations were carried out from 1974 to 1985 and later in 2000 and 2001. Illeta dels Banyets was an important commercial point until Middle Age, from where it was possible to dominate the maritime ways as well as the routes which connected the coast with the hinterland. The first prehistoric evidences of occupation dated back to the end of the Neolithic (c. 3000 BCE). Nevertheless, the most outstanding remains come from El Argar times. During the Early Bronze Age, two large cisterns partly excavated in the rocks were constructed. In its surroundings housing structures and several tombs were also discovered. Inside the tombs have been found metal awls and knives, ivory bracelets, pommels and buttons and some ceramic pots. The radiocarbon dates place the Argaric occupation between c. 1950 to 1550 cal BCE.

SITE	SAMPLE NUMBER	ARCHAEOLOGICAL CONTEXT	LAB ID	DATE BP	CALIBRATED DATA 2σ	AGE/SEX	PERIOD
Illeta dels Banyets	Ban2	grave I -449 (Caja 39)	Beta-188926	3360±50	1761-1510 BCE	Adult male	Early Bronze Age

References:
- López Padilla, J. A., Belmonte Mas, D., de Miguel Ibáñez, M. P., 2006. Los enterramientos de la Illeta dels Banyets de El Campello. Prácticas funerarias en la frontera oriental de El Argar. In J. A. Soler Díaz, ed. *La ocupación prehistórica de la Illeta dels Banyets (El Campello, Alicante)* MARQ, Serie Mayor, 5 Diputación de Alicante, pp. 119-171.

La Bastida (Totana, Murcia, Spain)

Lull, V., Micó, R., Rihuete-Herrada, C. & Risch, R.

La Bastida is a 4,5 ha hilltop site in the confluence of the mountain ranges of Tercia and Espuña and 3 km north from the fertile Guadalentín river floodplain. It was one of the first Bronze Age sites known in Spain, being excavated by different scholars since the late 19th century (Inchaurrandieta, 1875, Siret & Siret, 1887, Martínez Santa-Olalla et al., 1947, Lull et al., 2015). The current 'La Bastida Project' is carrying out extensive excavations and has explored over 6000 m^2 of the site since

2009 (Lull et al, 2014). As a result, data concerning 80 new architectural precincts and nearly 90 graves, as well as thousands of artefacts have been documented. With over one hundred ^{14}C dates La Bastida offers one of the best dated stratigraphies on the Iberian Peninsula (e.g., Lull et al., 2014; 2015b; 2015c). The site was inhabited along three phases between *ca.* 2200 and 1600/1550 cal BCE. Phase I (ca. 2200 – 2025 cal BCE) was characterised by small rounded huts made of mud and wooden posts, a few larger supradomestic buildings in stone and a monumental, complex and innovative fortification system unparalelled in Western Europe. During phase II (ca. 2025-1850 cal BCE) the site was fully reorganised according stone buildings covering the step slopes. Monumental structures were built on the summit, and a large water reservoir in the lower southeastern slope. The first graves are then documented. Nevertheless, most of the remains belong to Phase III (ca. 1850 to 1600/1550 cal BCE), when a population of around 1.000 people has been suggested inhabiting a extremely dense plan of terraced buildings that show differences in size and function (metallurgy, bone and textile production, grain processing and storage). Dozens of single or double inhumations in *pithoi* or cists under the occupation floors include a wide array of grave goods which point to social class asymmetries and differences related to biological sex. During the first half of the 2nd millennium cal BCE, La Bastida was the capital of a State polity in the context of a four-level settlement pattern, and one of the most influential Argaric centres, perhaps along with Lorca (Murcia) and El Argar (Almería). It was abandoned 1600/1550 cal BCE and never resettled on a permanent basis.

SITE	SAMPLE NUMBER	ARCHAEOLOGICAL CONTEXT	LAB ID	DATE BP	CALIBRATED DATA 2σ	AGE/SEX	PERIOD
La Bastida	Ba6-1	Ba 6-1	KIA-50633	**3298±27**	1631-1506 BCE	Adult male	Early Bronze Age
La Bastida	Ba12-1	Ba12-1	KIA-39254	3495± 26	1891-1745 BCE	Adult male	Early Bronze Age

References:

- Inchaurrandieta, R. de, 1875. Notice sur la montagne funéraire de La Bastida – Province de Murcie (Espagne). In *Congrés International d'Anthropologie et d'Archéologie Préhistorique, Copenhague* (1869), Imprimerie de Thiele, pp. 344-350.
- Lull, V., Micó, R., Rihuete-Herrada, C., Risch. R., 2014. The La Bastida fortification system: new light and new questions on the Early Bronze Age societies in the Western Mediterranean. *Antiquity* 340, pp. 395-410.
- Lull, V., Micó, R., Rihuete-Herrada, C., Risch. R., 2015a. *Primeras investigaciones en La Bastida (1869-2005)*. Arqueología Argárica – Proyecto Bastida 1, Integral, Murcia.
- Lull, V., Micó, R., Rihuete-Herrada, C., Risch. R., 2015b. Transitions and conflict at the end of the 3rd millenium BC in south Iberia. In H. Meller, H. W. Arz, R. Jung & R. Risch, eds. *2200 BC – A climatic breakdown as a cause for the collapse of the old world?*, pp. 365-407.
- Lull, V., Micó, R., Rihuete-Herrada, C., Risch. R., 2015c, When 14C dates fall beyond the limits of uncertainty: An assessment of anomalies in Western Mediterranean Bronze Age 14C Series, *Radiocarbon*, Vol 57, Nr 5, 2015, pp. 1029-1040.
- Martínez Santa-Olalla, J. et al.,1947. *Excavaciones en la ciudad del Bronce Mediterráneo II, de La Bastida de Totana (Murcia)*. Ministerio de Educación Nacional, Informes y Memorias nº 16, Madrid.
- Siret, H. & Siret, L., 1887. *Les Premières Âges du Métal dans le Sud-est de l'Espagne*. Antwerpen.

La Mina (Alcubilla de las Peñas, Soria, Spain)

Rojo Guerra, M.A.

The site La Mina was excavated between 2008 and 2011 by the Institute of Arcadia-General Foundation of Valladolid University (Rojo et al., 2014). It includes a classic passage grave with a corridor longer than five meters oriented south-southeast. The site suffered from a systematic process of closure and dismantling of its stone structure with all orthostats of the chamber and the passage being removed. However, the burial chamber, from which the remains of approximately 20 buried individuals have been recovered, remained intact. At the time of the burials, the appearance of the tomb was modified by increasing the diameter and height of the original mound, placing a menhir higher than three meters possibly on top of the monument, and building a wall of orthostats parallel to the old burial surrounding the entire perimeter wall. As a result, the original collective grave had been transformed into a ceremonial center, and given its unique topographic location the mound became a territorial landmark. A radiocarbon date from a human bone from the ossuary (Beta-316132, 4970±30 BP, 3893-3661 cal BCE, Rojo et al., 2015) places the tomb at the beginning of Megalithism in the inner Iberian Peninsula, at the early 4th millennium cal BCE.

SITE	SAMPLE NUMBER	ARCHAEOLOGICAL CONTEXT	PERIOD
La Mina	Mina1	Sample 1 skull 3	Middle-Late Neolithic
La Mina	Mina5	Sample 5 skull 9	Middle-Late Neolithic
La Mina	Mina7	Sample 7 skull 11	Middle-Late Neolithic
La Mina	Mina9	Sample 9 skull 13	Middle-Late Neolithic
La Mina	Mina10	UE 8 2404-2407 skull 15	Middle-Late Neolithic
La Mina	Mina14	UE 8 3437-3439	Middle-Late Neolithic
La Mina	Mina16	UE 8 2480-2482	Middle-Late Neolithic
La Mina	Mina17	UE 8 2408-2410	Middle-Late Neolithic

References:
- Rojo, M. et al., 2014. From the tomb to a Monument: the exceptional process of transformation of the megalithic grave of La Mina (Alcubilla de las Peñas, Soria). In M. Rojo, Ch. Scarre & C. Tejedor, coord. *Megalithic biographies: cycles of use and closure session. Burgos: XVII UISPP Congress (1-7 septiembre 2014)*. Oral communication.
- Rojo Guerra, M. A., et al., 2015. El tiempo y los ritos de los antepasados. La Mina y el Alto del Reinoso, novedades sobre el megalitismo en la cuenca del Duero, 2015. *ARPI Arqueología y Prehistoria del Interior Peninsular*. Vol 03-2015.

La Tarayuela (Ambrona, Soria, Spain)

Rojo Guerra, M.A.

The site La Tarayuela was excavated in 2002 by the Institute of Arcadia-General Foundation of Valladolid University (Rojo et al., 2005, 2010). It is a collective burial with a diachronic use. The remains of at least seventeen individuals in an area of around five meters were documented. There were many anatomical connections which suggest that at first the individuals were deposited in primary position, and the later use was the cause of the movements inside the ossuary and of the bones assemblages. The original structure of the tomb, that kept the ossuary, was probably built of

limestones and wood. It suffered a systematic process of closure by fire and dismantling of the rest of its building. Later the structure was covered with a large barrow of nine meters in diameter that closed and monumentalized the tomb. Afterwards, two pits were made in the mound, containing two individual burials, one of which has been dated. Two radiocarbon dates on charcoal (Bln- 5541, 5000±38 BP, 3943-3696 cal BCE; Bln- 5540, 4892±36 BP, 3764-3636 cal BCE) and other one on human bone (Kia- 27669, 4775±30 BP, 3643-3388 cal BCE) place the monument at the beginning of 4th millennium cal BCE (*ibídem*), an early moment in the process of the implantation of collective monumental burial in the lands of the inner Iberian Peninsula.

SITE	SAMPLE NUMBER	ARCHAEOLOGICAL CONTEXT	PERIOD
La Tarayuela	Tar2	UE 13 H82	Middle-Late Neolithic
La Tarayuela	Tar3	UE 15 F2-H1	Middle-Late Neolithic
La Tarayuela	Tar4	UE 7	Middle-Late Neolithic
La Tarayuela	Tar5	UE 14 RH-H56	Middle-Late Neolithic
La Tarayuela	Tar6	UE 13 bone 99	Middle-Late Neolithic
La Tarayuela	Tar7	UE13 H66	Middle-Late Neolithic
La Tarayuela	Tar8	UE 14 RH-H62	Middle-Late Neolithic
La Tarayuela	Tar9	UE 14 bone 84	Middle-Late Neolithic
La Tarayuela	Tar10	UE 13 H 108	Middle-Late Neolithic
La Tarayuela	Tar11	UE 14 Inventario 521	Middle-Late Neolithic

References:
- Rojo, M. et al., 2005. *Un desafío a la eternidad: tumbas monumentales del Valle de Ambrona*. Junta de Castilla y León: Conserjería de Cultura y Turismo, Arqueología en Castilla y León, 14
- Rojo, M, Garrido, R. & García, I., 2010. Tombs for the dead, monuments to the eternity: the deliberate destruction of megalithic graves by fire in the interior highlands of Iberia (Soria province, Spain)". *Oxford Journal of Archaeology*, 29(3), pp. 253-275.

Lorca (includes the sectors Los Tintes, Madres Mercedarias, Castillo de Lorca, Rincón de Moncada and Zapatería, Murcia, Spain)

Martínez, A. & Ponce Martínez, J.

The El Argar settlement of Lorca (Murcia) spreads over different locations under the present-day town, from the Medieval castle (Castle Sector) on the hilltop to the floodplain areas close to the Guadalentin valley. Most of the available information is the result of rescue excavations carried out during the last 30 years (Martínez-Rodríguez et al,. 1996, Fontenla et al,. 2004, Haber et al., 2008, Pujante & Martínez-Rodríguez, 2002, 2010). Stratigraphical and radiocarbon data show a continuous occupation during Argaric times (2200-1550 cal BCE) following an extensive but disperse Late Neolithic and Copper Age settlement. On the other hand, Argaric contextual remains correspond to domestic and productive areas and more than 50 tombs. If the scattered findings reported so far belonged to a continuous urban plan, Lorca would probably be the largest Argaric settlement reaching nearly 10 ha.

SITE	SAMPLE NUMBER	ARCHAEOLOGICAL CONTEXT	LAB ID	DATE BP	CALIBRATED DATA 2σ	AGE/SEX	PERIOD
Lorca	MMI3	Madre Mercedarias Iglesia Tomb 4-1	OxA-17056	3456±27	1878-1692 BCE	Adult female	Early Bronze Age
Lorca	Cast1a	Castillo de Lorca	-	-	-	Adult, sex nd	Early Bronze Age
Lorca	Rinc1	Rincón de Moncada 1	-	-	-	Adult male	Early Bronze Age
Lorca	Rinc2	Rincón de Moncada 2	-	-	-	Adult female	Early Bronze Age
Lorca	Tint1	Los Tintes	OxA-7667	3560 ± 35	2020-1773 BCE	Adult male	Early Bronze Age

References:
- Fontenla, S., Gómez Martínez, J. A. & Miras, M., 2004 Lorca, poblado más extenso y primigenio de la cultura del Argar. *Alberca*, 2, pp. 39-52.
- Haber, M., García-Ruiz, M. & Ramos, F., 2008. Rincón de Moncada (Lorca, Murcia). Ejemplo de evolución de rituales funerarios en un mismo espacio. In *Actas de las I Jornadas de Antropología Física y Forense.* Instituto Alicantino de Cultura Gil Albert, Alicante, pp. 57-65.
- Martínez Rodríguez, A., Ponce, J. & AYALA, M.M., 1996. *Las Prácticas funerarias de la Cultura Argárica en Lorca, Murcia*, Caja de Ahorros de Murcia y Ayuntamiento de Lorca, Lorca.
- Martínez, A. & Ponce, J., 2002. Segunda intervención arqueológica en la Plaza de Juan Moreno, nº 8, confluencia con calle los Tintes, Lorca. *Memorias de Arqueología* 10, pp. 150-160.
- Pujante, A., & Martínez Rodríguez, A., 2010. Los enterramientos argáricos de la excavación arqueológica en el convento de Madres Mercedarias de Lorca (Murcia). *Alberca*, 8, pp. 7-40.

Miquel Vives 69/73 (Terrassa, Barcelona)

Oliart, C. & Vicente, O.

The archaeological site of Miquel Vives 69/73 was excavated in 2002 by the Department of Prehistory of the Autonomous University of Barcelona. The site was partially destroyed during the building of an apartment block in the city of Terrassa, located at 40 km from Barcelona. Only a funerary pit was recovered during the archaeological work. The absolute radiocarbon date of a human remain from level 5 (KIA-35362, 3310±35: 1682-1505 cal BCE) confirms the dating of this funerary structure between the end of the Early Bronze Age and the beginning of the Middle Bronze Age (Vicente 2003; Soriano, 2013). The structure has a 1.25 m deep access well with a lateral funerary chamber of 1.30 cm length. The chamber was blocked with medium size stones (30-50 cm) during its last funerary use. The funerary deposit contained a primary burial and several secondary depositions of bones corresdponding to at least two different moments. Few fragments of pottery and two small lithic tools were also documented (Vicente 2003). The burial contains a minimum of 6 individuals (4 adults and 2 children). The children were 3-6 years and 7-9 years old. The adults correspond to at least 3 males and one female individual. This type of funeral structure is quite common between the late Chalcolithic and the Middle Bronze Age of the central Catalonian plain, with parallels at sites such as Can Roqueta, Can Ballarà, Can Filua or Bòbila Bonastre.

SITE	SAMPLE NUMBER	ARCHAEOLOGICAL CONTEXT	PERIOD
Miquel Vives	MIV1	Miquel Vives 39, Infans II (7-8 y.)	Early Bronze Age
Miquel Vives	MIV2	Miquel Vives 259, adult male	Early Bronze Age

References:
- Vicente, O. & Mameli, L. 2003. Memòria de la intervenció d'urgència al solar del carrer Miquel Vives 69/73 de Terrassa, Vallès Occidental. *Servei d'Arqueologia. Direcció General de Patrimoni Cultural. Departament de Cultura. Generalitat de Catalunya* [unpublished]
- Soriano, I. 2013. Metalurgia y sociedad en el nordeste de la Península Ibérica (finales del IV-II milenio cal ANE). *Oxford, Archaeopress, British Archaeological Reports International Series, 2502.*

Moita do Sabastião (Salvaterra de Magos, Portugal)

Araújo, A.C.

Moita do Sebastião (MS) is one of a series of Late Mesolithic shell middens located on the Muge – a small river that enters the Tagus on its left bank. The site was discovered in 1864 by Carlos Ribeiro; geologist of the Portuguese Geological Commission. It was subsequently excavated on several occasions, especially during the 1880s and 1950s; the latter directed by the French archaeologist, Jean Roche (Cardoso & Rolão, 2002-2003). Although severely damaged in 1952, it was possible to understand that Moita do Sebastião, dated to approximately 6030 – 5330 cal BCE (Lubell et al., 1994), was one of the most important and largest shell middens of the Muge complex. It has a residential character, which included several domestic features such as fire-places, post-holes and pits, etc. The lithic industries (including geometric armatures) and faunal remains indicate the exploitation of a wide range of terrestrial and aquatic resources. Although accumulated by the living, MS was also a place for the dead, who were buried in individual graves below the shell midden deposits. The minimum number of individual humans represented in the site is still controversial, but may have been as many as 100 (Jackes et al., 1997). Stable isotope data show that they had a diet comprising both marine and terrestrial foods in roughly equal proportions (Lubell et al, 1994).

SITE	SAMPLE NUMBER	ARCHAEOLOGICAL CONTEXT	LAB ID	DATE BP	CALIBRATED DATA 2σ	AGE/SEX	PERIOD
Moita do Sebastião	Seba1	22	To-131	7240±70	6235-5994 BCE	Adult female	Mesolithic

References:
- Cardoso, J. L. & Rolão M., 2002-2003. Prospecções e escavações nos concheiros mesolíticos de Muge e de Magos (Salvaterra de Magos): contribuição para a história dos trabalhos arqueológicos efectuados. *Muge - Estudos Arqueológicos*. 1, pp- 7-169.
- Jackes, M., Lubell, D., & Meiklejohn, C., 1997. Healthy but mortal: human biology and the first farmers of Western Europe. *Antiquity*, 71, pp. 639-658.
- Lubell, D. et al., 1994. The Mesolithic-Neolithic transition in Portugal: isotopic and dental evidence of diet. *Journal of Archaeological Science*, 21, pp. 201-216.
- Cunha, E. & Cardoso, F., 2002-2003. New data on Muge Shell middens: a contribution to more accurate numbers and dates. *Muge - Estudos Arqueológicos*, 1, pp. 171-183.

Molinos de Papel (Caravaca, Murcia, Spain)

Pujante, A., Fregeiro, M.I., Oliart, C. & C. Rihuete

Molinos de Papel was explored as a rescue excavation in 1999-2000 and 2007 under the modern town of Caravaca (Murcia) (Pujante, 2005, Marín et al., 2012). It is located on a flat area near to the Argos

River and dated to the Final Chalcolithic (Bell Beaker) and local non-Argaric Bronze Age. The most common archaeological features are pits of different shapes and sizes most of them interpreted as storage containers, rubbish pits, hearths or semisubterranean huts (*fondos de cabaña*) built with mud and wooden posts, although there are some examples with stone foundations. A few of these 'negative structures' had a funerary function containing single or double burials with grave goods related to the Bell Beaker tradition (ivory buttons, copper Palmela point). Radiocarbon dates associated to the double tombs 1 and 2 and to a single inhumation with a remarkable set of ivory objects date this site between *ca*. 2250 and 2050 cal BCE, that is, contemporary to the initial stages of the Argaric society developing only a few kilometers away.

SITE	SAMPLE NUMBER	ARCHAEOLOGICAL CONTEXT	LAB ID	DATE BP	CALIBRATED DATA 2σ	AGE/SEX	PERIOD
Molinos de Papel	Mop1	MP1039	MAMS-11826	3780 ± 30	2295-2060 BCE	Adult male	Late Copper Age
Molinos de Papel	Mop2	MP1059	MAMS-11827	3711 ± 29	2199-2027 BCE	Adult female	Late Copper Age
Molinos de Papel	Mop3	MP1065	MAMS-11828	3701 ± 26	2196-1985 BCE	Adult male	Late Copper Age

References:
- Lull, V., Micó, R., Rihuete-Herrada, C., Risch. R. 2015. Transitions and conflict at the end of the 3rd millenium BC in south Iberia. In H. Meller, H. W. Arz, R. Jung & R. Risch, eds. *2200 BC – A climatic breakdown as a cause for the collapse of the old world?*, pp. 365-407.
- Marín, J., López-Padilla, J. A., de Miguel, M. P., 2012. Un excepcional ajuar ebúrneo de los inicios de la Edad del Bronce en Molinos de Papel (Caravaca de la Cruz, Murcia). In, A. Banerjee, J.A. López-Padilla & Th. Schuhmacher eds. *Elfenbeinstudien 1: Marfil y elefantes en la Península Ibérica y el Mediterráneo occidental*. Deutsches Archäologisches Institut-Museo Arqueológico de Alicante, Alicante, pp. 157-171.
- Pujante, A., 2005. El yacimiento prehistórico de Los Molinos de Papel (Caravaca de la Cruz, Murcia). Intervención arqueológica vinculada a las obras de infraestructura del Plan Parcial SCR2, 1999-2000, *Memorias de Arqueología*, 14, pp. 133-172.

Monte Canelas, Hipogeu I, III (Portimão, Portugal)

Morán, E,; Silva, A. M. G.; Parreira, R.; Kunst, M.

At the site of Monte Canelas, located in the Algarve, circa 700 m north-nortwest of the Alcalar settlement, during the construction of modern houses there were found collective graves from the transition of the 4th to the 3rd millennium BCE. There are at least four rock-cut tombs (in Portuguese hipogeus). The analysed remains come from rock-cut tombs I and III. Hipogeu I was excavated meticulously between 1992 and 1994 (Parreira & Serpa 1995, 236-239; Silva 1996; Morán & Parreira 2004, 36-38; Neves & Silva 2010), with two interconnected chambers where it was possible to identify two phases of funerary depositions, separated by a partial collapse of the rock-cut vault. Osteological analysis identified a total MNI of 171 (Silva 1996). The first phase included a MNI of 147 individuals among which five burials survived 'in-situ'. They were interred in fetal position, in individual cells with grave goods (Silva & Parreira 2010). Until now, eight radiocarbon dates were obtained (Morán 2014; Silva, 1996) including one of a piece of charcoal (perhaps a rest of a torch) of the top level of the lowest funerary layer (ICEN-1149, 4460±110, 3497-2889 cal BCE) and seven from human bones. Six, were obtained from humans bones recovered from the lower funerary layer: a right tibia from an anatomic connection (OxA-5514, 4370 ± 60, 3326-2888 cal BCE), a left femur

(OxA-5515, 4420±60, 3336-2911 cal BCE) and long bones from the *in situ* burials (Beta-290365, 4970±40, 3927-3654 cal BCE) (Beta-290366, 4250±40, 3926-2680 cal BCE) (Beta-290368, 4450±40, 3338-2934 cal BCE) (Beta-305583, 4550±40, 3484-3100 cal BCE). And another one from a femur, from the upper funerary level (Beta-290364, 3620±30, 2117-1894 cal BC).

Hipogeu III was found during the excavation of a doorway to a modern house in construction (Morán et alii 2005; Morán & Parreira 2007, 85-86). The excavated part was a little bit more than 1 m^2, whereas the larger part of the tomb remains untouched for future excavations under the surface of the modern house's garden. The excavated part of the tomb contained rests of at least 13 individuals, seven of which were adults – men and women -, the youngest individual was approximately three years old (Silva et al. 2005). Some artifacts of amphibolite and flint were found, but nothing that could specify the chronology of the tomb. Until date there are no radiocarbon dates of this tomb, which possibly belongs more or less to the same period as Hipogeu I.

SITE	SAMPLE NUMBER	ARCHAEOLOGICAL CONTEXT	PERIOD
Monte Canelas I	Moca1	MCI 145.48	Early Copper Age
Monte Canelas I	Moca2	MCI 225.42	Early Copper Age
Monte Canelas I	Moca3	MCI 145.88	Early Copper Age
Monte Canelas I	Moca4	MCI 225.1	Early Copper Age
Monte Canelas I	Moca7	MCI 384.45	Early Copper Age
Monte Canelas I	Moca8	MCI 218.21	Early Copper Age
Monte Canelas III	MCIII2	172.54	Early Copper Age

References:
- Morán, M.E., 2014. El asentamiento prehistórico de Alcalar (Portimão, Portugal). La organización del territorio y el proceso de formación de un estado pristino en el tercer milenio a.n.e. Tesis Doctoral, Universidad de Sevilla.
- Morán, E. & Parreira, R., 2004. Alcalar 7. Estudo e reabilitação de um monumento megalítico, Cadernos 6. Lisboa.
- Morán, E. & Parreira, R., 2007. Alcalar. Monumentos Megalíticos, Roteiros da Arqueologia Portuguesa 10, Lisboa.
- Morán, E.; Parreira, R.; Sánchez Liranzo, O. (2005). Monte Canelas (Alcalar, Portimão): Trabalhos arqueológicos de salvamento de um habitat da época islâmica no Barrocal Algarvio. Xelb, Silves, 5, pp. 133-156.
- Neves, M.J. & Silva, A.M., 2010, L'hypogée funéraire de Monte Canelas I: révision des donnés et potentiel informatif. Antropo, 22, 11-18.
- Parreira, R. & Serpa, F., 1995. Novos dados sobre o povoamento da região de Alcalar (Portimão) no IV e III milénios A.C. In 1º Congresso de Arqueologia Peninsular, Actas VII (Porto, 12-18 de Outubro de 1993), Trabalhos de Antropologia e Etnologia 35, 3, Porto, pp. 233-256.
- Silva, A.M., 1996. Paleobiology of the population inhumated in the Hipogeu of Monte Canelas I (Alcalar-Portugal). Actas do XIII International Congress of Prehistoric and Protohistoric Sciences, Vol. III. Forlì, Itália, 8 - 14 September,: 437 - 446.

- Silva, A.M; Ferreira, MT.; Parreira, R.; Morán, E.; SánchezLiranzo, O.; Benisse, V., 2005. "Hipogeu III de Monte Canelas: análise dos restos ósseos humanos. Resultados preliminares". Colóquio Internacional Transformação e Mudança no Centro e Sul de Portugal, 3500 a2000 a.n.e. Cascais, 6 – 9 October.
- Silva, A.M. & Parreira, R., 2010, Hipogeu I de Monte Canelas: caracterização antropológica dos enterramentos in situ e das conexões anatómicas. In Gonçalves, V. S. & Sousa, A. C., ed., Transformação e Mudança no Centro e Sul de Portugal: o 4.º e o 3.º milénios a.n.e. Cascais: Câmara Municipal, p. 421-428.

Tholos de Pai Mogo I (Lourinhã, Portugal)

Kunst, M.; Silva, A.M.

Pai Mogo I (or Paimogo I) is a corbel-vaulted tomb on the Rio Grande, one km from the Atlantic coast. The site was discovered in 1968, with excavations carried out in 1971 during the month of October (Spindler & Gallay, 1972; Gallay et al., 1973). Two radiocarbon dates on human remains indicate the site was in use between 3100 BCE to 2500 BCE (Silva 2002). Little information is available about the context of the human remains recovered from this burial (Gallay et al. 1973) but these were found commingled, fragmented but good preserved. The MNI is 413, including 290 adults (Silva 2002, 2003). The tomb consisted of a more or less elliptic burial chamber (diameter of 4.85 m East-West and 4.5 m, North-South) and a corridor of 6.6 m length. An extensive array of objects includes Late Neolithic and Copper Age (or Chalcolithic) artefacts, such as decorated pre-Beaker and Beaker ceramics, groundstones and flaked stone tools, bone tools, limestone idols, other limestone objects, and copper implements (Spindler and Gallay 1972; Gallay et al. 1973). Two radiocarbon dates were obtained on human bones, which indicate the site was used between the end of 4th millennium to the middle of 3rd millennium (Sac-1556, 4250 ± 90 BP, 3095-2575 cal BCE, UGAM-22150, 4030 ± 25 BP, 2619-2475 cal BCE) (Silva, 2002).

SITE	SAMPLE NUMBER	SAMPLE	PERIOD
Pai Mogo I	Pai1	PM 12951	Late Neolithic/Chalcolithic
Pai Mogo I	Pai3	PM 12267	Late Neolithic/Chalcolithic
Pai Mogo I	Pai6	PM 12338	Late Neolithic/Chalcolithic
Pai Mogo I	Pai7	PM 12600	Late Neolithic/Chalcolithic
Pai Mogo I	Pai11	PM 12327	Late Neolithic/Chalcolithic
Pai Mogo I	Pai12	PM 3250	Late Neolithic/Chalcolithic
Pai Mogo I	Pai13	PM 12377	Late Neolithic/Chalcolithic
Pai Mogo I	Pai15	PM 12425	Late Neolithic/Chalcolithic
Pai Mogo I	Pai16	PM 12357	Late Neolithic/Chalcolithic
Pai Mogo I	Pai18	PM 12556	Late Neolithic/Chalcolithic
Pai Mogo I	Pai19	PM 12916	Late Neolithic/Chalcolithic
Pai Mogo I	Pai21	PM 12395	Late Neolithic/Chalcolithic

Pai Mogo I	Pai22	PM 12726	Late Neolithic/Chalcolithic

References:
- Gallay, G., et al., 1973. *O monumento pré-histórico de Pai Mogo* (Lourinhã) (Lisboa).
- Silva, A. M. 2002. Antropologia funerária e Paleobiologia das populações Portuguesas (litorais) do Neolítico final/Calcolítico. Unpublished PhD thesis, Coimbra, University of Coimbra.
- Silva, A. M., 2003. Physiological Stress in a Late Neolithic/Chalcolithic Portuguese Population: Tha Case of Paimogo I. In M. P. Aluja, A. Malgosa & R. M. Nogués, eds. *Antroplogía y Biodiversidad*. Barcelona, pp. 506-512.
- Sousa, A. C., 2016. Megalitismo e metalurgia. Os tholoi do Centro e Sul de Portugal. In A.C. Sousa, A. Carvalho & C. Viegas, eds. *Terra e Água. Escolher sementes, invocar a Deusa. Estudos em homenagem a Victor S. Gonçalves*, UNIARQ WAPS, Estudos & memórias 9, Lisboa, pp. 209-241.
- Spindler, K. & Gallay, G., 1972. Die Tholos von Pai Mogo / Portugal. *Madrider Mitteilungen* 13, pp. 38-108.

Gruta do Poço Velho (Cascais, Lisboa, Portugal)

Kunst, M.

Poço Velho is a natural cave, a karstic complex, with several entrances, A. Mendes called them Furnas 1 to 9. On the occasion of the 9th International Congress of Anthropology and Prehistoric Archaeology at Lisbon, there had been carried out three excavation campaigns in March, April and May 1879 under the direction of Carlos Ribeiro. The local director of these excavations was António Mendes. Until to date no documentation of theses excavations had been found, only three letters from A. Mendes to C. Ribeiro and some lists of the found objects. These had been put into boxes with labels. The labels show sometimes the date of the find and some explication of the find contexts. Most archaeological material comes from the Furnas 1, 2 and 3. In the following years several articles by different authors had been published on Poço Velho, and 1946 and 1947 a new investigation had been carried out by Abreu Nunes, publishes by A. do Paço (Paço, Bartholo & Brandão, 1959). Only in 2008 Víctor dos Santos Gonçalves published a monograph with 591 pages on the site collecting and discussing all information available to date and presenting all the found objects by excellent drawings and photographs, and he also let date by radiocarbon analyses 11 human bone collagen samples (Gonçalves, 2008). In conclusion the cave site of Poço Velho was used as cemetery in the end of the 4th (Beta-244394, 4520±40 BP, 3360-3097 cal BCE) and in the 3rd millennia BCE (Beta-245137, 4030±40 BP, 2834-2468 cal BCE). V. Gonçalves distinguishes especially three phases, 1st the Middle- and Final- Neolithic, 2nd an earlier Chalcolithic and 3rd a later Chalcolithic with the Bell Beaker phenomenon (Beta-178463, 3960±40 BP, 2575-2341 cal BCE) (Gonçalves 2008).

SITE	SAMPLE NUMBER	ARCHAEOLOGICAL CONTEXT	PERIOD
Poço Velho	Pove1	223.1912	Late Neolithic
Poço Velho	Pove2	223.1921	Late Neolithic

References:
- Gonçalves, V. S., 2008. As Ocupações Pré-Históricas das Furnas do Poço Velho (Cascais), *Colecção Cascais Tempos Antigos 3*.
- Paço, A. do, Bártholo, M. L. & Brandão, A., 1959. Novos achados arqueológicos das Grutas de Cascais, *Actas e Memórias do I Congresso Nacional de Arqueologia, Vo. 1*, pp. 147-159.

Valencina – Area 9 (Castilleja de Guzmán, Sevilla)

Kunst, M., Mejías, J.C., Cruz-Auñón Briones, R.

The archaeological excavation at Valencina - Area 9 in 2007 (Méndez, 2007) took place in the requirement to evaluate the archaeological potential of the lands affected by the PGOU of Castilleja de Guzmán. The area is part of the necropolis of Valencina de la Concepción, which has an extention of approximately 220 hectars, and which is separated by a large dich of 3 km length and differ from the prehistoric settlement, with an extent of approximately 240 ha (Mejías-García et al., 2015). At Valencina – Area 9 were documented about 29 graves (28 from the Chalcolithic with chamber and corridor of the tholos type, and 1 from the tartessic epoch (8th-7th centuries BCE); which are grouped such as following: 1 grave planking of schist slabs and delimited by a ditch of 25 m diameter and 1.5 m wide, 3 graves with masonry, 19 graves planking of schist slabs, 5 graves without a stone covering, and 1 grave from the tartessic epoch (Cruz-Auñón & Mejías-García, 2013). These 28 graves of the 3rd millennium BCE are only 13% of the 221 graves documented to date in Valencina de la Concepción. To these have to be added 43 depots of human remains in other types of structures inside of the settlement (ditches, pits, etc.) (Mejías-García et al., 2015). The tomb from which the samples were taken is the UE 171. It is a grave with chamber and corridor in bad conservation conditions, which only the chamber (2,35 m diameter) was conserved with scarce 0,12 m depth. In it were documented remains of 5 burried indivuduals, three of them in primary position, one with great anatomical absences and another one in a great disarticulation state (Méndez, 2007). These 5 individuals seem to be less than 1% of the 603 individuals counted in total to date at Valencina de la Concepción (Mejías-García et al., 2015). Only 4 of them were sampled, and gave positive results 3 of them: UE 1286, 1288 and 1290. The UE 1286 is an individual possibly male, about 18-25 years old; UE 1288 is an individual possibly male, about 25-35 years old; and UE 1290 is a female individual, about 20-30 years old (Pecero, 2007).

SITE	SAMPLE NUMBER	ARCHAEOLOGICAL CONTEXT	PERIOD
Señorío de Guzmán	Guz1	UE 1286	Copper Age
Señorío de Guzmán	Guz2	UE1288	Copper Age
Señorío de Guzmán	Guz4	UE 1290	Copper Age

References:
- Cruz-Auñón Briones, Mª R. & Mejías-García, J.C., 2013. Diversidad de prácticas funerarias e identidades en el asentamiento de Valencina de la Concepción (Sevilla). El asentamiento prehistórico de Valencina de la Concepción (Sevilla), pp. 175-199.

- Mejías-García, J.C. et al., 2015. Análisis del modelo de organización espacial de la necrópolis de Valencina. *Arqueologia de Transiçao: O Mundo Funerário. Actas do II Congreso Internacional Sobre Arqueologia de Transiçao*, pp. 52-70.
- Méndez Izquierdo, E., 2007: Memoria Preliminar de Intervención Arqueológica Puntual en Castilleja de Guzmán. Sevilla. Año 2007. (Memoria inédita).
- Pecero Espín, J.C., 2009: Memoria Final de Intervención Arqueológica Puntual en Castilleja de Guzmán. Sevilla. Informe antropológico. (Memoria inédita).

Tabayá (Aspe, Alicante, Spain)

Hernández Pérez, M. S., López Padilla, J.A & Jover Maestre, F. J.

This settlement is located on a hilltop at a strategic position between the middle and the lower valley of the Vinalopó River. It extends over just one hectare approximately. The occupation of the site seems to date back to the end of the Copper Age. At an early stage the settlement became part of the El Argar Culture, becoming a frontier placement between the Argaric territory and the so-called "Valencian Bronze Age" Culture. Excavations between 1987 and 1991 provided a deep stratigraphy and revealed a long sequence of occupation, including the Late Bronze Age. More than a dozen burials were discovered, mostly Argarics tombs. Two of them provided three radiocarbon dates around c. 1950- 1850 cal BCE.

SITE	SAMPLE NUMBER	ARCHAEOLOGICAL CONTEXT	LAB ID	DATE BP	CALIBRATED DATA 2σ	AGE/SEX	PERIOD
Tabayá	Tab2	grave 3 (1988) - Corte 10	KIA-38217	3557±26	2009-1777 BCE	Adult male	Early Bronze Age

References:

- Hernández Pérez, M. S. & López Padilla, J. A., 2010. La muerte en el Argar alicantino. El Tabaià como paradigma. In A. Pérez Fernández & B. Soler Mayor, coord. *Restos de vida. Restos de muerte. La muerte en la Prehistoria* Museu de Prehistòria de València, pp. 221-228.

Valdescusa (Hervías, La Rioja, Spain)

Alonso Fernández, C. & Jiménez Echevarría, J.

The archaeological site Valdescusa was excavated in 2008 by Cronos SC Arqueología y Patrimonio, under the direction of PhD Carmen Alonso. The archaeological excavation in the settlement highlights the stratigraphic and cultural synchrony bet-ween elements traditionally considered Bell Beaker and Middle Bronze Age, acting as spatiotemporal link between regional synthesis of La Meseta y el Alto-Medio Valle del Ebro, where the chronological dimension of the phenomenon is very uneven. In both sides of the Cordillera Ibérica and especially in the corridor Alto Duero-Alto Ebro (provinces of La Rioja, Burgos, Soria and Navarra), concentrate atypical archaeological records outside the timeframe established for the peninsular Bell Beaker period. A radiocarbon date from a human bone (Ua-36345), places the burial E-45 in the Middle Bronze Age. Here are documented 41 domestic and funerary structures belonging to a “pits field”. Among the domestic structures exist pits-dumps, post holes and ditches were usually appears Bell Beaker materials and other productions of "smooth ceramic horizon" own of regional Middle Bronze Age. Among the funerary structures five

individual burials in pit are counted, primary deposits of three infants and secondary deposits in juveniles and adults. They all try to burials away from schemes tombs of ancient Bell Beaker. The dating on radiocarbon and thermoluminescence from the pit E-45 warn of the contemporaneity of the buried individual and the Bell Beaker ceramic materials accompanying inside the fill deposition. A double occupancy and the intrusive nature of these materials are discarded, being present in almost all archaeological structures. The pottery decorated with Bell Beaker motifs represents 10% of the total record in Valdescusa, a percentage that can hardly explain their high symbolic value because to be associated with an everyday household use; is included within the 'Silos style', variety of Ciempozuelos.

SITE	SAMPLE NUMBER	ARCHAEOLOGICAL CONTEXT	LAB ID	DATE BP	CALIBRATED DATA 2σ	AGE/SEX	PERIOD
Valdescusa	Vald1	E 45	Ua-36345	3400 ± 35	1866-1616 BCE	Adult male	Middle Bronze Age
Valdescusa	Vald2	E 47	-	-	-	Child	Middle Bronze Age
Valdescusa	Vald3	E 69	-	-	-	Child	Middle Bronze Age
Valdescusa	Vald4	E 74	-	-	-	Juvenile	Middle Bronze Age
Valdescusa	Vald5	E 77	-	-	-	Juvenile	Middle Bronze Age

References:
- Alonso Fernández, C. & Jiménez Echevarría, J., 2015. La progresión del 'fenómeno' Campaniforme durante el Bronce Medio en el entorno del Sistema Ibérico: el poblado de Valdescusa (Hervías, La Rioja, España). *Munibe Antropología-Arkeología* 66, pp. 147-162.

Valle de las Higueras, Cueva 3 (Huecas, Toledo, Spain)

Bueno-Ramírez, P., Barroso-Bermejo, R. & de Balbín-Behrmann, R.

Cave 3 is one among 10 hypogea structures documented in the site of Valle de las Higueras, Huecas, Toledo, by the team of Universidad de Alcalá de Henares. It is a necropolis found in the southern hillside of one of the elevations that shape this area. It is the usual topography found on the wide valleys of the tributary rivers of river Tajo.The architecture of the hypogea variates significantly. They are made of soft limestone from the geologic formation. The later was chipped from the exterior to the interior, work that can be seen in both chambers of Cave 1. Walls are covered with thin limestone stones and mud. There is an internal division on the burials of chambers and niches, with small accesses from the main corridor sometimes even marked. These chambers and niches draw easier or more complex plans, like the hypogea documented on the South-east of the Iberian Peninsula. On each one of the structures decorated pottery has various combinations, though none of them is mixed with metal, associated with non-decorated pottery, nor exotic ornaments, such as amber or variscite. Specifically Cave 3 had a main chamber, where M3 was found, and a secondary one, accompanied by three niches on the rear end. In these three niches children, two located on niche 3a and the rest on niche 3b, a woman, in niche 3b, and two males, in niche 3, were found together with Ciempozuelos Bell Beaker pottery (Beta-157730, 3810±40 BP, 2456-2137 cal BCE). They were all dated to the second half of the third millennium cal BCE, thus being contemporaneous. The main chamber of Cave 3 was occupied in the same chronology as the niches (Beta-205141, 3869±40, 2466-

2208 cal BCE) the same as the secondary one. Nonetheless it is important to notice a slight difference in the remains treatment. In the secondary chamber (Beta-157732, 3830±40 BP, 2458-2148 cal BCE) human remains were primary deposited, buried in fetal position and probably inside sacks. On the other hand those in the main chamber were buried in supine position. M3 was found on the superior area of the deposit. He was an adult male with evident signs of arthrosis in the kneecaps. He was placed unnaturally, as his legs were bended over his torso. Grave goods were placed in an organised manner; non decorated vessels were accumulated in the south-east wall, containing food and beverage remains. In the opposite wall vessels, copper burins and flint arrowheads were as well perfectly organised. Among the individuals buried, only one woman of the earliest remaining had personal adornment. Feasting rituals similar to those found in dolmens from the south-east of the Iberian Peninsula are confirmed through the finding of beer, mead, fish stew or pork fat in pottery vessels.

SITE	SAMPLE NUMBER	ARCHAEOLOGICAL CONTEXT	PERIOD
Valle de las Higueras	Hig2	Cave3c Ind.3	Late Copper Age

References:
- Barroso Bermejo, R. Et al., 2014. Nekropolen des 3. Jahrtausends v. Chr. im Zentrum der Iberischen Halbinsel. *Madrider Mitteilungen*, 55, pp. 1-28.
- Barroso Bermejo, R., et al., 2015. Enterramientos individuales y enterramientos colectivos en necrópolis del megalitismo avanzado del interior: la cueva 9 de Valle de las Higueras. *IIº Congresso Internacional sobre Arqueologia de Transição: O Mundo Funerário.* British Archaeological Reports International 2708, pp.165-176.
- Bueno Ramirez, P., Barroso Bermejo, R. & de Balbín Behrmann, R., 2000. Valle de las Higueras (Huecas, Toledo, España). Una necrópolis Ciempozuelos con cuevas artificiales al interior de la Península. *Estudos Pré-históricos* 8, pp. 49-80.
- Bueno Ramirez, P., Barroso Bermejo, R. & de Balbín Behrmann, R., 2005. Ritual campaniforme, ritual colectivo: la necrópolis de cuevas artificiales del Valle de las Higueras, Huecas, Toledo. *Trabajos de Prehistoria* 62, pp. 67-90.
- Bueno Ramirez, P., Barroso Bermejo, R. & de Balbín Behrmann, R., 2007. Campaniforme en las construcciones hipogeas del megalitismo reciente al interior de la Península Ibérica. *Veleia* 24/25, pp.771-790.
- Bueno Ramirez, P., Barroso Bermejo, R. & de Balbín Behrmann, R., 2011.Entre lo visible y lo invisible: registros funerarios de la Prehistoria reciente de la Meseta Sur. Arqueología, Sociedad, territorio y paisaje. *Biblioteca Praehistórica Hispana* 28 (Madrid 2011),pp. 53-73.
- Bueno Ramirez, P., Barroso Bermejo, R. & de Balbín Behrmann, R., 2012. *5000 años atrás. Primeros agricultores y metalúrgicos del valle de Huecas (Huecas, Toledo)* Toledo .
- Bueno Ramirez, P., Barroso Bermejo, R. & de Balbín Behrmann, R., 2012. Les grottes artificielles et le Campaniforme à l'intérieur de la péninsule ibérique: la nécropole de Valle de las Higueras. Sépultures collectives et mobiliers funéraires de la fin du Néolithique en Europe occidentale. *Archives d'Écologie Préhistorique.* Toulouse, pp.359-381.
- Bueno, P., Barroso, R. & de Balbín, R., 2013. Interior regions and places of collective memory: the megalithism of the interior basin of the Tagus, Iberian Peninsula. A reflection after reading of the Tara project, en M. O'sullivan, C. Scarre y M. Doyle (eds): *Tara from the past to the future*. Wordwell, pp.484-501.
- Bueno Ramirez, P., Barroso Bermejo, R. & de Balbín Behrmann, R., 2016. Between east and west: Megaliths in the center of the Iberian Peninsula. In Laporte, L. & Scarre, C. eds. *The Megalithic Architectures of Europe* (UK 2016).Oxbow Monographs, pp. 157-166.
